# Deep Learning Using Isotroping, Laplacing, Eigenvalues Interpolative Binding, and Convolved Determinants with Normed Mapping for Large-Scale Image Retrieval

**DOI:** 10.3390/s21041139

**Published:** 2021-02-06

**Authors:** Khadija Kanwal, Khawaja Tehseen Ahmad, Rashid Khan, Naji Alhusaini, Li Jing

**Affiliations:** 1 School of Computer Science and Technology, University of Science and Technology of China, Hefei 230009, China; khadijakanwal@mail.ustc.edu.cn (K.K.); husaini@ustc.edu.cn (N.A.); 2 Department of Computer Science, Bahauddin Zakariya University, Multan 60800, Pakistan; tehseen@bzu.edu.pk; 3 Department of Electronic Engineering and Information Science, University of Science and Technology of China, Hefei 230009, China; rashidkhan@mail.ustc.edu.cn

**Keywords:** deep learning, CNN, image retrieval, image content analysis, BoW, color image retrieval

## Abstract

Convolutional neural networks (CNN) are relational with grid-structures and spatial dependencies for two-dimensional images to exploit location adjacencies, color values, and hidden patterns. Convolutional neural networks use sparse connections at high-level sensitivity with layered connection complying indiscriminative disciplines with local spatial mapping footprints. This fact varies with architectural dependencies, insight inputs, number and types of layers and its fusion with derived signatures. This research focuses this gap by incorporating GoogLeNet, VGG-19, and ResNet-50 architectures with maximum response based Eigenvalues textured and convolutional Laplacian scaled object features with mapped colored channels to obtain the highest image retrieval rates over millions of images from versatile semantic groups and benchmarks. Time and computation efficient formulation of the presented model is a step forward in deep learning fusion and smart signature capsulation for innovative descriptor creation. Remarkable results on challenging benchmarks are presented with a thorough contextualization to provide insight CNN effects with anchor bindings. The presented method is tested on well-known datasets including ALOT (250), Corel-1000, Cifar-10, Corel-10000, Cifar-100, Oxford Buildings, FTVL Tropical Fruits, 17-Flowers, Fashion (15), Caltech-256, and reported outstanding performance. The presented work is compared with state-of-the-art methods and experimented over tiny, large, complex, overlay, texture, color, object, shape, mimicked, plain and occupied background, multiple objected foreground images, and marked significant accuracies.

## 1. Introduction

Nowadays artificial intelligence (AI) and machine learning (ML) based convolutional neural network (CNN) research has fascinated significant consideration. Due to its exceptional performance, CNN is applied in various fields—including image detection, image retrieval, and image classification [1]. AlexNet [2] was a good breakthrough and then VGGNet [3] was developed for large scale classification. GoogLeNet [4] applies combined inception modules including concatenation operations, convolutions with various scales, and pooling. Recently, ResNet [5] was introduced with more than 100 convolution layers. The number of trained weights are increased with number of increasing layers. This requires a huge amount for computation and significant computational time in training and the classification steps. The classification for these networks in the real time applications is also a challenging task. To overcome this problem, graphics processing units (GPUs) are normally applied to speed up the classification and training time [6]; however, a Tensor Processing Unit (TPU) is an equally good choice in non-parallel computing environment [7]. GPUs uses 1000 s of Arithmetic and Logic Units (ALUs) to compute heavy parallel and matrix processing neural networks. TPU do not contain general purpose hardware due to its domain specific architecture and normally adopted for massive multiplications and additions.

In practical applications, the classification acceleration is a main factor which is obtained by utilizing the power and storage resources [8]. The CNN architectures are employed for highest level of abstractions using deep learning architectures that are combined for many non-linear transformations [9]. State-of-the-art performance is achieved using CNN for various applications, including object recognition [10] and speech recognition [11]. CNN architectures have been presented to improve the workflow of image retrieval [12,13]. Furthermore, the features extraction by using deep learning methods have taken developments in the processing of multimedia contents. The CNN features [14] have attained high effectiveness in different retrieval and computer vision tasks. The combination of local features—such as color, shape, and texture—is performed with CNN features by incorporating scale invariant feature transform (SIFT) to detect the potential image contents. This is interested phenomena that exposes the combination of deep learning features with local features to find the background and foreground objects.

Image retrieval is a process of image extraction based on salient image features including shapes, texture, colors, and objects. Image retrieval system focuses on foreground and background image features with their semantic interpretation and spatial aspects. Therefore, content-based image retrieval is an intelligent image extraction system that retrieves the results based upon the actual image contents as contrast to text based retrieval [15]. Versatile local features have been proposed in last decades to support object recognition tasks and content-based image retrieval (CBIR). These local features includes SIFT [16] and SURF [17] methods to efficiently match local structures along with thousands of deep features being extracted. Six feature extraction techniques are extensively applied including image segmentation, color features, shape features, texture features, interest point detectors and combination of visual features. Image segmentation is applied to extract similar regions. For this, the common techniques are contour-based [18], thresholding [19], region growing segmentation [20], grid-based [21], *k*-means clustering [22], watershed segmentation [23], texture-based segmentation [24], statistical model [25], and normalized cut [26]. To focus the color representation, it is better achieved by applying different color models and spatial aspects. Color moments are also applied to match color similarity and indexing. Color space technique is used to convert color bins having their representational frequency. The color histogram of multiple objects with similar colors are also focused with large image databases. Color histogram has a problem to ignores main information like shape and texture. For image analysis, image quantization is attained in RGB space with pixel classification as interior or border pixel localization with four neighbors. In this regard, texture features analysis with their neighbor is not an easy task and rough, irregular, smooth, and random results. For this, the corner edges and similarities techniques are applied to model texture features.

In [27], texture features are extracted by incorporating different techniques which are invariant to shape [28] and rotation in [29]. The spatial texture features face complex indexing and searching process. It applies spectral texture methods for image transformation on frequency image domain using spatial filter banks. The feature extraction is achieved on transformed domain using statistical tools. Consequently, the techniques for spatial filter banks are vigorous to noise and makes prominent than spatial texture features. The fast Fourier transform (FFT) is also applied for spectral analysis to obtain spatial results [30].

The novelty of the presented method is the application of local features in combination with the strength of GoogLeNet, ResNet-50 and VGG-19 architectures for effective features extraction methods. The proposed method introduces a novel technique using Gaussian filters, isotropic filtering, multi-scale filtering, LOG (Laplacian of Gaussian), convolutions, derivatives, scale spacing, Eigenvalue computations, and feature reduction. The presented approach applies Gaussian filters whose results are purified with Laplacian of Gaussian. To produce the results on which Isotropic filtering is applied that builds the foundation for multiscale filtering. The multiscale filtering is applied to obtain the deep image filtering at various abstract levels. These features are aggregated with gray level convolution features which are concatenated with first order partial derivatives. To obtain the best texture values, Eigen coefficients are computed for these signatures. At an elementary level, BoW kernel function is applied on which non-maximum suppression (NMS) is performed to create the basis for interpolation of corner responses. Determinants are required to compute data points which are already produced by interpolated corner responses. After the computation of determinants, again convolution and derivatives are applied where the difference is that now second order of derivatives are applied instead of first order derivatives to obtain better differences. On this research, Laplacian of Gaussian (LOG) approximation is applied for scale spacing. These features are representative of image objects. On parallel, RGB channels are computed with the selection of color coefficients and normalized values mapping to get the color features. At this step, texture, objects and color features are extracted which are then concatenated with the CNN based trained feature vectors. The final assembly of feature vectors is the representation of textures, objects, colors CNN based image features for all strong image content candidates. Finally, for image retrieval the BoW architecture is applied to retrieve relevant image. The image conversion is performed from color to gray levels 0 to 255 and second level of normalization is applied for all channels, and integrated with produced signatures for presenting the massive feature vectors compactly. Principal component analysis (PCA) is used for redundant feature vectors. The GoogLeNet, ResNet-50, and VGG-19 architectures return the feature vectors for every image for which deep features have already been fetched. The GoogLeNet, ResNet, and VGG-19 based feature vectors are fused with deep features extracted from presented technique. These powerful features are combined to find the tiny, large, complex, overlay, texture, color, object, shape, mimicked, plain background, and multiple-object-based images. These image feature vectors are used as input to the BoW for efficient image indexing and image retrieval. To test the competitiveness of the presented method, it is experimented on ten standard image benchmarks such as Cifar-100, ALOT (250), Oxford Buildings, Cifar-10, FTVL Tropical Fruits, Corel-1000, Fashion (15), Caltech-256, Corel-10000, and 17-Flowers which contains millions of images from versatile semantic groups. The presented work has following novelties and contributions:

1. It comprehensively analyses and collects image contents such as color, object, texture, shape, and spatial information which produces significant recall and precision rates.
2. To attain better improved results, a method is introduced that improves the capabilities of ResNet-50, VGG-19, and GoogLeNet architectures using its internal coupling.
3. A light-weight description model and feature detection is presented that efficiently and effectively retrieves the related images from cluttered and complex databases.
4. First time proposed a technique that performs multilevel scaling, suppression, interpolation, determinants and scale spacing collectively to attain the detail of deep finer image contents.
5. Presented a color and gray level features based retrieval system to encompass the image features for edge, corner detection capabilities with color carrier candidate features.
6. The presented technique introduces a methodology that effectively returns significant performance on similar textures, overlay ambiguous objects, tiny objects, resize images, mimicked, color dominant arrangements, cropped objects, cluttered patterns, overlay ambiguous objects, color dominant arrangements, and similar textures.
7. A storage, computation and time efficient image retrieval system is presented that retrieves the images in fraction of time.
8. A new idea is presented that strengthens the normalized scaled features with BoW architecture for quick classification and indexing.

The remaining article is organized as follows. The related work about CNN with deep learning is presented in Section 2. The presented methodology is explained in Section 3. In Section 4, the experimental results which presents with graphs and tables, also discusses the results of experiments. In Section 5, the conclusion of the presented method is discussed.

## 2. Related Work

The Convolutional Neural Networks (CNN) have been researched by many existing methods to achieve highest performance for different applications. The recent trends are focused on understanding the Deep Neural Networks (DNN) performance for different complexity levels. In [31], a novel technique is presented to show the minor changes in results of image classification. Moreover, networks can be improved by adding new layers, and by contributing new computer vision algorithms [32]. The researchers introduced a feature extraction technique for image retrieval and image representation using CNN in [33]. The experimentation is performed on Cifar-100 and Cifar-10 benchmarks. For image classification using deep networks, a PCA approach is proposed in [34]. In [35], factorization is performed on similar pairwise semantic matrices into hash codes approximation for training images. Moreover, for efficient and effective image retrieval, CNN is used to make binary hash codes in [36]. Karhunen–Loeve transform (KLT) is used in discrete cases to present the stochastic processes in proper conditions [37]. An effective algorithm is presented in [36] for fast calculation for KLT operator. This fast KLT calculates eigenvectors efficiently when this is treated with small samples. In [38], deep learned features are computed by finding the symmetry in FAST scores with neighborhood, smoothing, and standard deviation. Feature scaling, reduction, and filtering is applied to resize the features for a variety of datasets. This approach indicates the potential research outcomes if it is compared with existing ResNet architecture. 

A fusion technique presents the ResNet, VGG, and GoogLeNet models [39] with an interconnection. The SVM and random forest techniques are applied for image classification. The experimentation is performed to achieve better performance on Standford 40 actions. In [40], an approach is proposed that is based on deep learning using five stages including image pre-processing, pre-trained CNN, semantic segmentation, query analysis, and image retrieval for NTCIR13Lifelog-2 dataset. The stemming for image labels from deep neural networks (DNN) in [41], researchers are applied AlexNet [3] and GoogLeNet for object recognition on ImageNet dataset. Moreover, the AlexNet, ResNet [5], GoogLeNet, and VGG [3] architectures are used for scene recognition on places365. The histogram of oriented gradients (HOG) method is employed to detect number of person in each image. In [42], a new technique is performed for comparison and dissimilarities of latest CNN algorithms including AlexNet, VGGNet, GoogLeNet, and ResNet on BelgiumTS benchmark. In addition, an object detection technique is presented to find better processing speed and object recognition ratios among CNN. Symmetric solution by using SVD, PCA, VGG, and Gaussian mixture model (GMM), and highest dimensional feature extraction, feature selection, region segmentation, and softmax respectively in [43]. Furthermore, feature selection for SVD and PCA using FC layers validated the accuracy of images classification.

Various algorithms and methods are proposed for feature extraction, description and detection, and object detection and classification. Research literature is shared to induce the similarity among object detection, filter banks, and texture filters. The implementation of semantic concept this technique is relatively hard, and difficult to understand the overlapped and cluttered objects. Therefore, the previous approaches are focused on image classification and single object detection for feature set [44]. The researchers in [45] focused their research on lower level feature extraction, and object recognition, including filter banks, HOG, GIST, and the bag of feature (BoF) applied using word vocabulary. 

The content-based image retrieval (CBIR) is used visual features including image edge, name suitability, color, and texture in input images [46]. The CNN is applied for image classification to retrieve images using cosine similarity. The CNN approach has demonstrated successful image retrieval tasks based on image classification. Many researchers contributed to the image processing task, the semantic gap among the lowest level of image features. In [47], a robust technique of CNN and sparse representation was proposed. Moreover, a novel technique is presented with an in-depth feature extraction using CNN, and increased image retrieval accuracy and speed using sparse representation. In this method is tested for image retrieval on MPEG7, ALOI, and Corel databases. Recent research has been deploying CNN for various types of object-based image classification. However, how to efficiently take advantage of deep CNN features with a trained network to increase the object-based retrieving images still required more research. 

The presented method is formulated with best suited approach for efficient image retrieval. The Gaussian filters whose results are purified with LOG. Multi-scale filtering is used after Gaussian filtering in presented technique. Derivatives and Eigenvalues are also used in presented method. NMS is performed to create the basis for interpolation of corner responses. LOG approximation is applied for scale spacing. CNN is applied to reduce features, and the image classification is performed by ResNet-50, VGG-19, and GoogLeNet. Spatial mapping and L2 normalization are applied to normalize images for searching and indexing purposes. The GoogLeNet, ResNet-50, and VGG-19 based feature vectors are fused with the deep features extracted from the proposed method. These powerful features are combined to find out overlay, texture, color, object, shape, mimicked, plain, complex and occupied background, multiple objected foreground images from the challenging image benchmarks. BoW is applied for efficient image retrieval and index. The presented method shows outstanding performance for all challenging benchmarks including Cifar-10, Caltech-256, Corel-1000, Oxford Buildings, Cifar-100, FTVL Tropical Fruits, Corel-10000, 17-Flowers, ALOT (250), and Fashion (15).

## 3. Methodology

**Figure 1:**
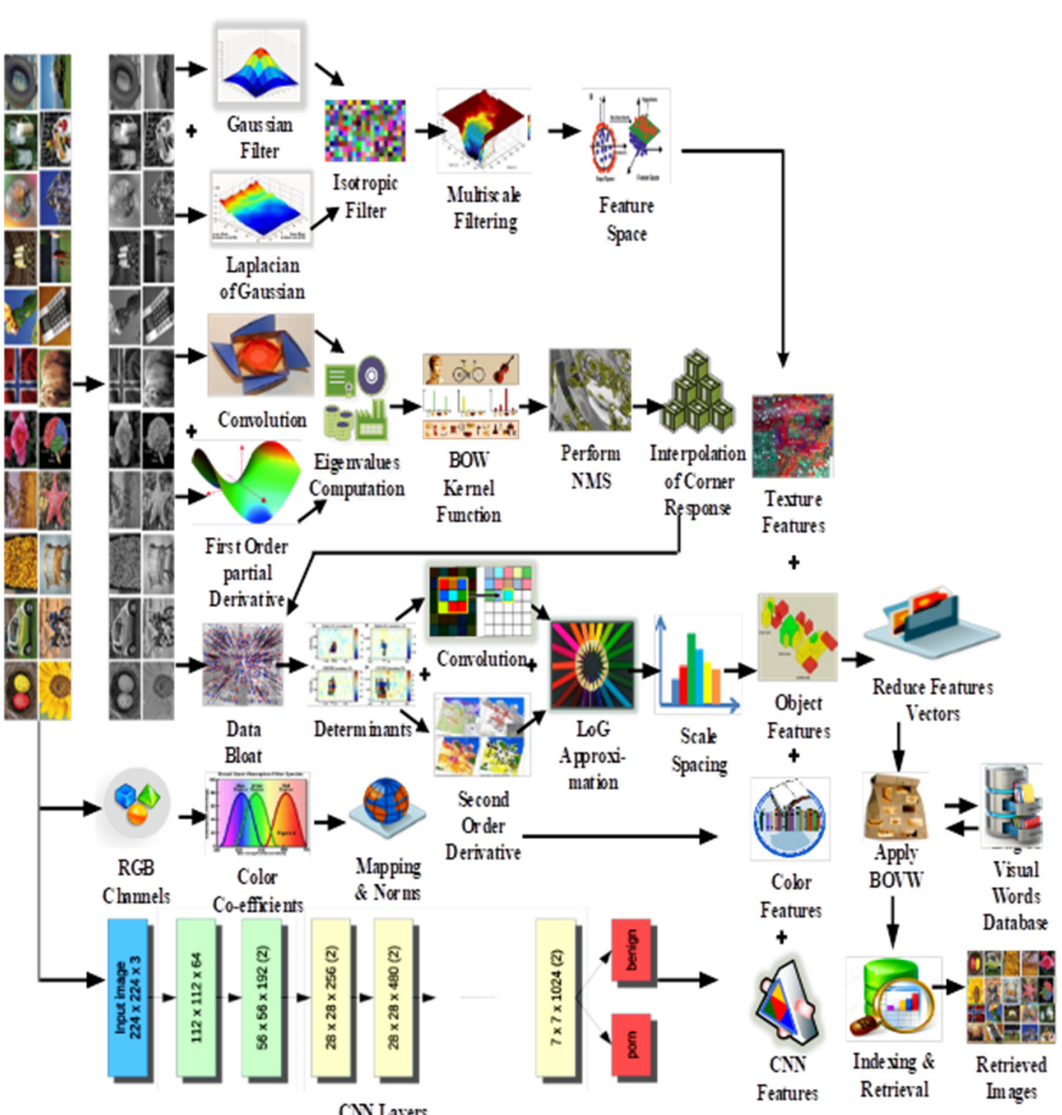
Step-by-step process of object detection.

In Figure 1, the presented architecture inputs an image and converts it into gray level to extract Gaussian, Laplacian, Isotropic, and scale-based features mapping. Moreover, the convolution and derivations are encapsulated of Eigen values to input for bag-of-words architecture to show the separation effects with interpretation. In parallel, data blots with determinants are computed at second order with LOG and convolved values. These feature vectors are fused and assembled with RGB maps, colors, norm features and concatenated with ResNet-50, VGG-19, and GoogLeNet separately for quick indexing and retrieval. 

### 3.1. Image Channeling

At the first and foremost step, is to find out the key points in the image. For this, image conversion is performed from color to gray levels 0 to 255 and that is input for first level of processing. The noise is removed from gray scale image by applying primitive techniques such that the gray scale image is formed with high and low intensity represented by white and black levels.

### 3.2. Isotroping and LOG Filtering with Derivations

In this step, The MR8 filter bank [48] is applied with 38 filters over eight responded filters to obtain texture orientation, neighboring and patterns. The arrangement of these 38 filters consist of eight Laplacian of Gaussian (LOG) filters along with four Gaussian filters to form 12 filters to be applied at three scales and generates 36 filters. These 36 filters are concatenated with first and second order of Gaussian to form 38 filter bank. To achieve the rotational invariance, the filters are employed at different orientations and scales. At each scale, the maximum response among multiple orientations is preserved. The final response at each position is an eight-dimensional feature vector having three scales for bar filters and edge, plus two for isotropic [49]. The filters comprise Gaussian, and Laplacian of a Gaussian (LOG) filter at scale *φ* = 10, a bar (second derivative) and also an edge filter of first derivative at six orientations and similar three scales respectively (*φ_h_*, *φ_p_*) = {*i*, *j*, *k*} where *i* = (1,3), *j* = (2,6) and *k* = (4,12). To obtain equal insensitive and mono directional context, isotroping is applied on the determined filters responses. These are collapsed at every scale due to filter responses through orientations using eight responses of filters to ensure rotational invariance in filter bank. Moreover, the filter bank of MR4 [50] is employed (*φ_h_*, *φ_p_*) = *k* scale [51]. MR4 contains 14 filters in which four are responded to obtain neighboring, pattern, and texture orientation. This classifier is used to complete the local neighbor values for pixel filter responses. The small neighborhoods with 3 × 3 size over to 7 × 7 attain higher categorization performance for multiple scale filter banks. It is important to distinguish between texture classes referred as joint classifier. The texture pattern analysis is a potential carrier for background and neighborhood similarities and helpful in semantic interpretation based upon texture distributed. Equation (1) defines for Markov random field (MRF) [51]

*γ*(*W*(*h_e_*)|*W*(*h*), *∀_h_* = *h_e_*) = *γ*(*W*(*h_e_*) | *W*(*h*), *h*∈*Ŧ*(*h_e_*)) (1)

In Equation (1), *h_e_* is used as a site in two-dimensional integer lattice, *W* represent image and *Ŧ* (*h_e_*) is the neighborhood of the site. It is defined *Ŧ* as *Ŧ* × *Ŧ* the square neighborhood. The value of central pixel is significant but its distribution must coordinate to its neighbors. To test this distribution conditioned, retain classifiers on the feature vectors from the set of *Ŧ* × *Ŧ* neighborhoods with left out central pixel. Classification ratios for *Ŧ* = 5 are improved when left out the center pixel and slightly worse for cases of *Ŧ* = 3 and *Ŧ* = 7. The joint distribution is sufficient to validate MRF model of the textures in dataset. It is an explicit model containing *γ*(*W*(*h_e_*)|*W*(*h*), *h*∈ *Ŧ* (*h_e_*)). At this point, textons are used to define central pixel joint probability density function (PDF) conditioned and their neighbors. The center pixels are applied for feature vectors in *Ŧ*_2_ − 1 dimensional space by similar dictionary for *q* textons for every *m* textons is putative elementary units of texture where *q* = 610, one-dimensional distribution of central pixels learns by t bin histogram. The joint PDF is represented by *m* × *t* matrix. Furthermore, a Gaussian filter property is that it is an operator which satisfying uncertain relation in Equation (2) [52]

(2) $${∆}_{z\;}{∆}_{v\;}\geq \frac{1}{2}$$

In Equation (2), ${∆}_{v}$ and ${∆}_{z}$ are variances in frequency and spatial domains respectively. Also, this property is allowed the Gaussian operator for provide best tradeoff among conflicting localization areas in frequency and spatial domains. The two-dimensional Gaussian filter uses rotational symmetric filter; that is a separate coordinate. The separability is significant for efficient computation when applying operation of smoothing using convolutions in the spatial domain. Moreover, the optimal smoothing filter is localized for both frequency and spatial domains for images, thus satisfies the uncertain relation which is given in Equation (2). Consider two dimensions Gaussian operator defines in Equation (3) [52]

(3) $$s\left(z,r\right)=\;\frac{1}{2\;\left(3.14\right)\varphi^{2}}\;e^{-\left(z^{2}+r^{2}/2\varphi^{2}\right)}$$

In Equation (3), φ is used for standard deviation and (*z*,*r*) are Cartesian coordinates for image. Gaussian filters are applied for various scales to image in Hildreth and Marr, a set for images with various smoothness levels is obtained. For image edges detection, it is also essential to find zero-crossings for the second derivatives. The Laplacian of a Gaussian (LoG) function is used as filter in Equation (4) [52]

(4) $$\begin{array}{ll} \nabla^{2}s\left(z,r\right) & =\;\frac{d^{2}}{dz^{2}}\;s\left(z,r\right)+\;\frac{d^{2}}{dr^{2\;}}\;s\left(z,r\right)\\ & =\;\frac{z^{2\;}+\;r^{2}-2\varphi^{2}}{2\left(3.14\right)\varphi^{6}}e^{-\left(z^{2}+r^{2}/2\varphi^{2}\right)} \end{array}$$

In Equation (4), it is an operator of independent orientation and the scale is assumed by φ. It is broken down at locations, at curves, and corners, the image intensity function changes in non-linearity manner beside an edge. In Algorithm 1, it computes the square neighborhoods for the intensity values which are already joints in step-1 and step-2. Then, it computes the central pixel with Ŋ × Ŋ neighbor which is classified for odd numbers in step-3 and step-4. The feature vectors are computed for Ŋ^2^ − 1 for which ң and ω bin are represented in step-6 and step-7. Finally, the co-occurrence representation is shown for filter banks and probability is computed in step-8 to step-10.
**Algorithm 1***Neighborhood computations algorithm*.
   Step-1: Square neighborhood preferences calculation   Step-2: Jointly introduce the intensity values   Step-3: Compute Ŋ × Ŋ neighborhoods with excluding central pixel   Step-4: Test classification for Ŋ = 3,5,7   Step-5: Include central pixel and retest step 2–5   Step-6: Form Ŋ^2^ 1 feature vector   Step-7: Show in ң values ω bin representation   Step-8: Compute co-occurrence representation of filter banks   Step-9: Compute joint probability P (İ)   Step-10: Compute conditional probability   P (Ϛ (y_^_)|Ϛ (Ή (y_^_))

### 3.3. Eigen, Harris, Gaussian Coefficients Composition

At this step, the KLT [37] and Harris algorithms [53] are applied to identify corner pixels of an image. The first order Gaussian derivative kernel approximates using box kernel to speed up the algorithm on which convolution is computed at with less resources on integral images. The integral images of *u*^2^*_x_*, *u*^2^*_y_*, and *u_x_u_y_* are used to increase the computation of cornerness response. The cornerness concentrates computation, and time consumption for Harris algorithms. Furthermore, the *D*(*γ*) cornerness of pixel is estimated by adding squares, and multiplies gradients with integration window *N* such as shown in Equation (5). Both algorithms measure the alteration in intensities because shifts the *N* for each directions are given points in the cornerness response of corner pixels are defined as in Equation (5) [53]

(5) $$D(\gamma )\;=\;\sum_{h\in N}\left\{\left[\begin{array}{cc} u_{h}^{2}\left(H\right) & u_{h}\left(H\right)u_{p}\left(H\right)\\ u_{h}\left(H\right)u_{p}\left(H\right) & u_{p}^{2}\left(H\right) \end{array}\right]\;\times \;£\;\left(H\right)\right\}\;=\;\left[\begin{array}{cc} U_{hh} & U_{hp}\\ U_{hp} & U_{pp} \end{array}\right]$$

with,

(6) $$u_{i}=\varphi_{i}\;(u⊗W)\;=\;(\varphi_{i}u)\;⊗\;W,\;i\;\in \;h,p),\;u=U\;(h,p;\;\phi )$$

Here, £ (H) is used for weighted function, *γ* is for central pixel, *N* is used for the integration window centralized at *γ*, *W* is an image, *u* is used for two dimensional Gaussian function, and *u_h_* and *u_p_* are gradients for image attained using convolution for Gaussian first order partial derivatives for *h* and *p* directions in Equation (5). The Harris corner detector is used to evaluate cornerness for each pixel without explicit decomposition of eigenvalues as described in Equation (7) [53]:

(7) $$A\;=\;\left|D\right|\;-\;ƙ\;\times \;(\mathrm{Trace}\;(D){)}^{2}$$

with,

(8) $$\left|D\right|\;=\;{ƛ}_{1}\times \;{ƛ}_{2}\mathrm{and}\;\mathrm{Trace}\;(D)\;=\;{ƛ}_{1}\;+\;{ƛ}_{2}$$

where ƛ_1_ and ƛ_2_ are eigenvalues of *D*. The value of ƙ is taken between 0.04 to 0.06. The image pixel is like a corner if the eigenvalues are large, the resultant *A* in a peak response.

The *D*(*γ*) eigenvalues are calculated by KLT. It is selected for points that computed the minimum eigenvalue as defined in Equation (9) [53]

*A* = ƛ_min_ = min (ƛ_1,_ ƛ_2_) (9)

(10) $${ƛ}_{\min}=\;\frac{1}{2}\left(U_{\mathrm{hh}}+U_{\mathrm{pp}}\;-\;\sqrt{\left(U_{hh}-U_{pp}\right)+4\;\times U_{hp}{}^{2}}\right)$$

The KLT and Harris algorithms uses similar method to detect the corner points, the difference is only the cornerness functions that is estimated in Equations (7)–(10) respectively. The KLT and Harris algorithms are divided in three steps as:

(a) The Gaussian derivatives for image *W*, *u_h_*, and *u_p_* are calculated using convolution with kernel of Gaussian derivative;
(b) The *D*(*γ*) matrix and cornerness measure *A* evaluate individually of every pixel; and
(c) The NMS and Quick-sort are applied to suppress local low points.

The complexity reduces with two aspects. First, the integral image reduces the complexity for both evaluation and convolution of cornerness response. Second, the efficient NMS adopts of NMS, then avoid high complex sorting. 

### 3.4. First Ordering, Kernel Approximation, and Recursive Filtering

At this stage, detection of corner feature point is required for computation of Gaussian first order partial derivatives, *u_h_* and *u_p_*, in directions of *h* and *p* with image *W* respectively. Moreover, the Gaussian kernels should be discrete and approximated with finite instinct response filter. It is required at least *4ϕ* filter length. The Gaussian derivative kernel results improved achievement of the Harris corner detector using better repeatability. The Gaussian derivative kernel is applied recursively for fast computations of convolutions. The Gaussian derivative filter is estimated with infinitely impulse of response filter [54] using recursive filter approach, which is allowed to set the length of filter. Using this method, computation for Gaussian derivative kernel of various scales are applied in time constantly. In SURF [55], first order Gaussian partial derivative kernel with box kernel is approximated. The gray areas are set as 0 in it. Also, the white and black areas are estimated using +1 and −1, respectively. The gradients are computed at low computation cost and also in constant time with integral image. Finally, only seven operations and eight memory accesses need to calculate gradient. Furthermore, the filter response normalizes with the size of filter. The convolution using Gaussian derivative kernel integrates two steps including low-pass filtering using differentiation and Gaussian. Next, Gaussian function is approximated using a triangle function subsequently integration with integral image method. Furthermore, the operations number are essential for box kernel that is independent for kernel size, then linearly increase with the kernel size. The multiplication operations reduce to 1 and other multiplications replace by subtraction and addition operations. 

### 3.5. Multiple Gradients Responses

The cornerness step is the most intensive in computation and also time consumption for KLT [37] and Harris algorithms [53]. The cornerness *D*(*γ*) for pixel evaluates by adding squares and multiplies of gradients in *N* as in Equation (5), which evaluates at each pixel. Therefore, the computation overlapping among pixels is lying in *N* occurs. It creates integral image for gradients such as in Equation (5), accelerating computation for corner response. The following Equations (11)–(13) are defined in [53]

(11) $$\;jj_{hh}\left(h,p\right)=\;\underset{h^{\prime }\leq h,\;p^{\prime }\leq p}{\sum }u_{h}^{2}\;\left(h^{\prime },\;p^{\prime }\right)$$

(12) $$jj_{pp}\left(h,p\right)=\;\underset{h^{\prime }\leq h,\;p^{\prime }\leq p}{\sum }u_{p}^{2}\;\left(h^{\prime },\;p^{\prime }\right)$$

(13) $$jj_{hp}\left(h,p\right)=\;\underset{h^{\prime }\leq h,\;p^{\prime }\leq p}{\sum }u_{h}\;\left(h^{\prime },\;p^{\prime }\right)h_{p}\left(h^{\prime },\;p^{\prime }\right)$$

From Equations (11)–(13), the integral images are created like summations *U_hh_*, *U_pp_*, and *U_hp_* in Equation (5), evaluated at low computational cost with four memory accesses and three operations. The repeated summation and multiplication operations in *N* from image of each pixel is exchanged with one-time creation for integral image; and after that easy operations of subtraction and addition. Furthermore, no loss is observed for detector efficiency with modification and contribution using huge speedup. The speedup is achieved by additional memory access for *jj_hp_* which not exist in original algorithm. The *u_h_u_p_* can be computed at same time for the present CPU internal registers and gradients read from memory, that is not for *W* as it is located and pre calculated in the memory.

### 3.6. Perform Non Maximal Suppressions with Corner Responses

In this step, the NMS is performed over cornerness response images that preserves a single location of each corner feature point. NMS is applied in two steps [56] for the KLT detector. Firstly, the quick-sort is performed of arranging *A* cornerness response in descending order over image [56,57,58], that is a more expensive operation computationally as sorting for the list of response points corresponds with image size. The non-maximal points are blocked using selective robust response points from response sorted list and eliminating low strong response points consecutive in list inside the distance *ƈ*. Finally, a lowest distance among feature points are required. NMS naive employment of local neighborhood for (2*ƈ* + 1) × (2*ƈ* + 1), each cornerness response is leading a highest Harris complexity [59]. Comparison is performed for all pixels lying in integration window (2*ƈ* + 1) × (2*ƈ* + 1) to find the maximum point. The maximum point is chosen if it is above threshold and greater than all pixels. This procedure is repeated for each pixel in cornerness response image. In [60], the efficient non maximum suppression (E-NMS) is introduced for efficient extraction of single feature locations for each corner region. The E-NMS is performed NMS in image blocks rather than pixel by pixel, then reduces the complexity of computation. Moreover, the minimum distance enforcement and quick-sort steps are mostly intended for tracking [56] a unique location for each corner region. Furthermore, it switches order by applying the effective NMS, that is low complex computationally, and sort the feature points as cornerness response. The sorting performs on a small number of points, complexity reduces extremely. The E-NMS algorithm works as follows. First, it partitions the image in size blocks (*ƈ* + 1) × (*ƈ* + 1). Secondly, the maximum element is found within each individual block. If it passes the test of local maximum and existing threshold, then the feature point location is retained. For the KLT algorithm, sorting is performed for the feature points as cornerness response is related with them. In Algorithm 2, interest points are detected by applying non-maximal suppression on neighborhoods with their variants and determinants as shown in step-1 to step-3. After this, interpolation is computed at different scale factors for which the neighborhood extraction is performed based on contents description in step-4 to step-6. Similarly, variant extraction is computed based on gradients in step-7 and result in Haar wavelet response in step-8. Algorithm 3 shows Haar wavelet response by applying square region with their orientation and splits up in step-1 to step-3. Space based information is preserved with 5 × 5 boxes for which horizontal and vertical directions are computed and increased interval in step-4 to step-7. Geometric aspects are computed with deformation in step-8 and localization in step-9. Finally, results in weighted centered interest points to represent the feature vectors in step-10 and step-11.
**Algorithm 2***Find Interest Points algorithm*.
   Step 1: Apply cube 3 NMS neighboring   Step 2: Compute variants   Step 3: Separate the determinants   Step 4: Apply interpolation   Step 5: Apply scaling and image spacing   Step 6: Distribution content description ↔ neighboring extraction   Step-7: Gradient ↔ variant extraction   Step-8: Compute first order Haar wavelet response**Algorithm 3***Haar wavelet response effects algorithm*.
   Step 1: Square region formation   Step 2: Orientation selection   Step 3: Region split up   Step 4: Spatial information preservation   Step 5: 5 × 5 regularly spaced samples   Step 6: Compute $K_{x}$ for horizontal directions   Step 7: Compute $K_{y}$ for vertical directions    Step 8: Increase interest point orientation   Step 9: Deformation ← geometric aspects   Step 10: Localization ← geometric aspects   Step 11: Result ← weighted centered interest points

### 3.7. Hessian Blobs Detection

In this step, blob structures are detected by locations for determinants calculation. Consider a point *Z* = (*h*, *p*) for an image *W*, Hessian Matrix *U* (*h*, *φ*) of *h* at the scale *ɵ* is represented such as in Equation (14) [55]

(14) $$U(h,\varphi )\;=\;\left[\begin{array}{cc} l_{hh}\left(h,\;\varphi \right) & l_{hp}\left(h,\;\varphi \right)\\ l_{hp}\left(h,\;\varphi \right) & l_{pp}\left(h,\;\varphi \right) \end{array}\right]$$

In Equation (14), $l_{hh}\left(h,\;\varphi \right)$ is convolution for Gaussian second order derivative $\frac{\partial^{2}}{\partial h^{2}\;}\;$*z*(*φ*) and same for $l_{hp}(h,\;\varphi$) and $l_{pp}(h,\;\varphi$) with image *A* of point *h*. Gaussians are used for the optimal analysis of scale-space [61]. Moreover, Gaussians are used for discretization and cropping. The resultant is the repeated image rotated around of odd multiples $\frac{3.14}{4}$ which is the weakness of Hessian detectors. The repeatability is computed using multiples of $\frac{3.14}{2}$ due to square shape filter. LoG approximations are second order derivatives and evaluated with low computation cost by integral images. The nine square box filters are applied to Laplacian of Gaussian and presented at low scale for computing the blob response maps. These are represented by *l_hh_*, *l_pp_*, and *l_hp_*. The weights are applied to rectangular regions for easy and efficient computation, as in Equation (15) [55]

*Determinant* (*Lapprox*) = *lhh lpp* − (*wlhp*)^2^ (15)

In Equation (15), the weight *w* for filter responses is applied of balancing Hessian’s determinant expression. Moreover, filter responses normalize according to their size. This is guaranteed as a constant Frobenius normalization for some filter size and also used for analysis of scale space. The approximation of Hessian determinant is represented for blob response of an image at the location *h*. The responses are kept in map form for blob response over various scaling level and detected the local maxima. These scale spaces are applied as an image pyramid. The repeated images are smoothen using Gaussian and the sub-sampled according to highest level for pyramid. In [62], Lowe subtracts pyramid layers to make difference for Gaussians of images to find blobs and edges. The box filters can apply for each size at exact similar speed on the real image. This scale space is used to analyze the increase in filter size. Next, the following layers are managed using image filtering along with applying gradually higher masks. The divided scale space is represented as a series of filter response maps attained by convoluting similar input image by increasing filter size. Moreover, each octave is sub-divided to constant number for scale levels. Integral images have discrete nature, the difference of minimal scale among two consequent scales is depended on length *l*_1_ for negative and positive lobes of second order partial derivative in derivation direction (*h* or *p*), that is set as size length of third filter. Moreover, for 9 × 9 filter, the length *l*_1_ is 3. It is necessary to increase the size by the minimal of two pixels to obtain uneven size and to assure the existence of central pixel for two successive levels. Re-scaling mask presents round off errors. The errors are smaller than *l*_1_, this type of approximation is conventional. The construction of scale space initiates with 9 × 9 filter, that computes the blob response for image of smallest scale. Algorithm 4 shows the intermediate response and summed up operations in step-1. All the absolute values are computed and polarity with intensity is calculated in step-2 and step-3, respectively. The differentials for the sub-regions are computed and their aggregate is scale factor with descriptor in step-4 to step-7.
**Algorithm 4***Intermediate response and summed operation algorithm*.
   Step-1: Compute sum of absolute values   Step-2: Check polarity intensities   Step-3: Compute $\left|V_{x}\right|$ and $\left|V_{y}\right|$
      Where V contains differentials   Step-4: $s_{V}$
←
$\sum V_{x}$, $\sum V_{y}$
   Step-5: Apply s to all sub-regions   Step-6: Sum Ǵ = $s_{1}$ + $s_{2}$ + $s_{3}$ +---------+$s_{n}$
   Step-7: scale factoring ← descriptor ← unit vector

### 3.8. Spatial RGB Mapping

The presented approach introduces a new way of presenting color channels by their coefficients to present the contents of color image in efficient and compact way. For this, a color histogram is captured to show the color distribution. The spatial correlation for changing the color is presented by distance in presented approach. Let *£* be an image, *i* colors in *£* are quantized such as *x_£_*,…*x_i_*. Color represents a pixel *Q* = (*a*, *k*) can be described in Equation (16) [63]

(16) Q=(a,k) ∈ £

For computation of distance between pixels *Q*_1_ (*a*_1_, *k*_1_) and *Q*_2_ (*a*_2_, *k*_2_) in Equation (17) [63], we define

(17) $$\left|Q_{1}\;-\;Q_{2}|\;≜\;max\;\{|(a_{1}\;-\;a_{2})|,\;|k_{1}\;-\;k_{2}|\right\}$$

In Equation (18) [63], the color histogram *Y* of an image *I* and *n* ∈[i], we define as

(18) $$Y_{\mathrm{dn}}(£)\;≜i^{2}.Pr\;\left[Q\in £x_{n}\right]\;Q\in \;£$$

In Equation (18), *Y_xn_ (W)/i*^2^ returns the probability of pixel color *Wx_n_* where *W* is an image and *x_n_* is a pixel color. The histogram *Y* is a linear function in image size and it can be calculated as time O($i^{2}$). Let the distance *x* ∈ [*i*] is used to fix a priori. The correlogram for the image *£* is represented of *a*,*k* ∈ [*i*]; *k* ∈ [*x*] in Equation (19) [63]

(19) $${\;Ή}_{x_{a}x_{k}}^{l}\left(£\right)≜Pr\;\left[Q_{2}\in £x_{a}\;||Q_{1}-Q_{2}|=P\right]\;Q_{1}\in £\;x_{k}\;\;Q_{2}\in £$$

Equation (19) is used to define the spatial arrangement for color pixels in image. Ή is represented by the probability and color pixel $x_{k}$ at distance *P* that is away from a given color pixel in Equation (16). Then, the spatial relationship among similar color values is presented in Equation (20) [63]

(20) $${ȵ}_{x}^{P}(£)≜Q_{x}^{P}.x^{\left(£\right)}$$

Equation (20) derives from Equation (18), where ȵ is denoted by probability of *x* color pixel and *p* is used for distance.

### 3.9. Covariant Selection for Detection

The presented approach shows compact image features; however, to reduce image retrieval time, the features are presented with their coefficients using PCA. After performing different scaling level steps, the proposed approach applies the algorithm to reduce features which applies various mathematical designs to reduce irrelevant images and apply compression on images [48]. The principal component analysis is used to reduce variables, and these coefficients are applied for the measurement of number of factors which are not correlated [64]. The PCA is applied in situation where most of variables are measured for same construct [64] and also applied to process data comprising extraction of few synthetic variables; that is named as principal components. The principal components are sequence of data projection. PCA is employed to compress and reduce dimension for finding high variance based coefficients [64]. Let *m* be represented by dimensions on a vector *v* of $q_{1}$ random variables and reduce measurement from $q_{1}$ to s [65]. The principal component analysis is applied to find out the linear combinations such as $a_{1}^{\prime }v,\;\;a_{2}^{\prime }v\;\ldots \;a{\;}_{r}^{\prime }v$ that have an extreme data variance focuses on un-correlated with last $a_{1}^{\prime }ve$. To solve maximization problem, eigenvectors are $a_{1},\;a_{2},\ldots ..,a_{r}$ of covariance matrix *e* that corresponds to *s* largest eigenvalues. Furthermore, the eigenvalues show variances of principal components respectively and summation results for first *r* eigenvalues to sum of variances for every $q_{1}$ real variables that is denoted by proportion for each variance in real database, applied for *s* principle components [65]. 

### 3.10. Image Retrieval and Indexing Using BoW

In this step, the bag-of-words (BoW) architecture is used of quick image retrieval and indexing. In BoW architecture, each image is denoted with single linear vector. First, BoW model applies the controls for local feature descriptor as Scale Invariant Features Transform (SIFT) [16]. Second, BoW comparison of single vector is applied using dis-similarity score that is simply employed. The SIFT descriptor is represented by patches like numerical vectors. The SIFT is created to collect equal sized dimensional vectors having 128 bits that is represented by one byte for compact and effective image representation. The occurrence count of each visual word is represented in form for histogram of each image. An inverted image index generates efficient image retrieval based on histogram. Each index is represented by one visual word where small parts of an object carry versatile information—including shape, color, and texture. Visual words contain pixel changes with respect to low level features, descriptors and filters. An image identity list also creates to map terms using images. Finally, image ranking is employed to count visual words number shared between indexed images and query image. The highest number for shared words of image takes its rank to the top. The BoW model is not captured the location, spatial information and co-occurrence of visual words. In the presented approach, the spatial color extraction technique is embedded spatial information for feature vectors (FV) at feature extraction time that is the results of more relevant retrieved images.

### 3.11. Deep Image Networks

The presented method employs the architecture of deep convolutional neural network that is compared with ResNet-50, GoogLeNet, and VGG-19 to check the affectivity and accuracy of presented approach. The presented method uses inception-v1 [4], that is also called GoogLeNet, is a deep lightweight network such as its basic concept is improved performance and efficient computation. The GoogLeNet is relatively less computation cost is a product of two concepts: (1) optimum CNN with sparsity as introduced in [66]; and (2) the dimension reduction with 1 × 1 convolutional layer as presented in [67]. The Inception-v1 components in GoogLeNet architecture are used three filter sizes including 5 × 5, 3 × 3 and 1 × 1 and also a max-pooling layer. For dimension reduction, the 5 × 5 and 3 × 3 filters precedes with 1 × 1 convolutional layer, while the max-pooling layer succeeds with 1 × 1 convolutional layer. GoogleNet architecture designed for efficient computation and reduced the number of parameters. GoogleNet architecture is 22 deep layers when counts layers with parameters and 27 layers when counts pooling. In independent building blocks, there are 100 layers which are used for GoogLeNet architecture. Alternatively, VGG-19 architecture is employed to test its strength that primarily depends on the CNNs model. The VGG-19 employs 16 layers or 19 layers because of its simplicity like 3 × 3 convolutional layers are fixed on the top for increment with the depth level. In VGG-19, for reduction of volume size, the max pooling layers are applied as a handler. Two FC layers are applied with 4096 neurons. In the training phase, the convolutional layers are employed for feature extraction and the max pooling layers connected with some convolutional layers for reduction of feature dimensionality. In the first convolutional layer, 64 kernels were used for the feature extraction from input images. Fully connected layers were applied to prepare the feature vector (FV). The PCA is used to reduce dimensionality and the features selection for image with better results of classification. It is a significant task to reduce the highly dimensional data using PCA. Ten-fold cross validation is used to categorize the DR images depend on softmax method in the testing phase [43]. The performance of presented technique using VGG-19 is used to compare with other features extraction models including GoogLeNet and ResNet-50. Moreover, the presented method uses ResNet-50 architecture to fuse with the proposed feature extraction and detection to achieve maximum accurate results. The presented approach adopts second level non-linearity. The dimensions are essential of equal size as *i* and *Ŗ* in *Y = Ŗ* (*i*, {*W_j_*}) + *i*. The output and input channels are used to apply changes and a linear estimation $W_{s}$ of shortcut connection performs to match the dimensions in Equation (21) [5]

(21) $$Y=Ŗ\;(i,\;\{W_{j}\})\;+\;W_{t}I$$

In Equation (21), the identity mapping appropriates the degradation problem and $W_{t}$ is employed for matching dimensions. *Ŗ* (*i*, {*W_j_*}) is used to represent multiple convolutional layers. 

Using ResNet-50, GoogLeNet and VGG-19, performance of many applications for computer vision have been increased including objects detection and recognition. The feature vectors are fused with the GoogLeNet, ResNet-50 and VGG-19 generated feature vectors to create a powerful image signature that deeply represents the object and shape features.

## 4. Experimentation

### 4.1. Databases

The effective and accurate image retrieval (IR) system is experimented on a variety of suitable image databases. Many contributions are essentially domain oriented. Various images datasets are applied to their object information, complexity, spatial color, generic usage of CBIR, versatility, and object occlusion. Experimentation is performed by selecting standardized benchmarks including Cifar-100 [68], Fashion (15) [69], Cifar-10 [68], (250) [63], Corel-1000 [63], FTVL Tropical Fruits [63], Corel-10000 [63], Oxford Buildings [70], Caltech-256 [71], and 17-Flowers [63]. These challenging databases exist to a wide range of semantic images groups. The effectivity and accuracy of results are affected using image characteristics including occlusion, cluttering, size, quality, object location color, and overlapping. The selected datasets characteristics are in different range of images, image classes and classes have various objects types located at foreground and background [72].

#### 4.1.1. Input Process

The system takes a query image that is generally a color image. The conversion is performed on colored image into gray scale 0 to 255 levels for presented algorithm and this converted image is used as input image to CNN. The input image is used from image datasets in input process. In presented work, the input image is selected from Cifar-100, Corel-1000, Cifar-10, ALOT (250), Corel-10000, Oxford Buildings, Fashion (15), FTVL Tropical Fruits, 17-Flowers, and Caltech-256. For the query image, features are extracted, and the bag-of-words architecture is employed to find k-nearest images and index. The robustness and superiority of presented approach is capable to classify the shapes, colors, textures, and objects. The images are tested for testing and training with 30% and 70% proportions of testing and training respectively. The random images selection is performed from each image category using permutation.

#### 4.1.2. Recall and Precision Evaluation

The two metrics, recall and precision are applied for evaluation of performance accuracy. The calculation of true positive ratio is used as recall and predicted positive values are employed to precision. Then, precision is calculated for each image category using Equation (22) [72] and recall is calculated for each image category using the following Equation (23) [72]

(22) $$\mathrm{Precision}=\frac{E_{w\left(n\right)}}{E_{u\left(m\right)}}$$

(23) $$\mathrm{Recall}=\frac{E_{w\left(m\right)}}{E_{o}}$$

where $E_{w\left(m\right)}$ is applied at query image to retrieve the related images, $E_{u\left(m\right)}$ represents the contrast of query image and aggregates of the available relevant images.

#### 4.1.3. Mean Average Precision Evaluation

The mean average precision (mAP) is mean precision that is computed over all queries. The mAP is described as in Equation (24) [73]

(24) $$\mathrm{mAP}\;=\;\frac{\sum_{q=1}^{l}E\left(q\right)\times rel\left(q\right)}{r}$$

In Equation (24), *E*(*q*) is denoted by average precision for top *q* image retrieval ratios; a binary indicator function *rel* (*q*) is equal to one if qth image retrieved rates are related to current query and otherwise zero; and *r* and *l* represent number of related results for current query and summation of retrieved images results respectively. 

#### 4.1.4. Average Retrieval Precision Evaluation

The graphs of average retrieval precision (ARP) indicate ARP for presented approach of different databases. The ARP is calculated for every category by applying Equation (25) [63]

(25) $$\mathrm{ARP}\;=\;\sum_{b=1}^{i}AQ_{b}|\;i$$

The *AQ* is applied for the average precision and *b* is applied of all categories. The ARP is employed to calculate *AQ* for all classes of every dataset. The ARP graphs are used to show data placement in order where each data bar is denoted by corrected number of retrieved images regardless of the class. The x-axis represents number of categories contrary to *AQ*. The *AQ* is decreased slowly when increased number of classes. The reason is that the large number of classes plot a big denominator. The ARP is calculated for databases such as Cifar-100, Cifar-10, Corel-1000, Oxford Buildings, ALOT (250), Fashion (15), Tropical Fruits, Corel-10000, Caltech-256, and 17-Flowers.

##### 4.1.5. f-Measure Evaluation

The calculation of f-measure is performed as harmonic mean (HM) for recall (*t*) and average precision (*s*) as described in Equation (26) [74]

(26) $$f\;=\;\frac{2\times s\times t}{s+t}$$

Here, *f* is applied for f-measure in Equation (26). Also, *t* is used for recall and s for precision. 

### 4.2. Discussion and Results

The experimentation is performed on Core i7 machine (GPU) with 8 GB RAM. MATLAB R2019a is used for experimentation using CNN and image processing toolboxes. The strong and versatile deep image and layer functions are integrated in code-base to reveal the image. Moreover, the computational efficiency and execution time to retrieve images and are computed against inverted file indexing and non-hierarchical searching.

To test the efficiency of the presented technique, the experiments are performed on Cifar-100, Corel-1000, Cifar-10, 17-Flowers, ALOT (250), Corel-10000, Oxford Buildings, Fashion (15), Caltech-256, and Tropical Fruits datasets. The versatility and superiority for presented approach is tested on three different convolutional neural networks including GoogLeNet, VGG-19, and ResNet-50. 

#### 4.2.1. Performance on the Cifar-10 Dataset

**Figure 2:**
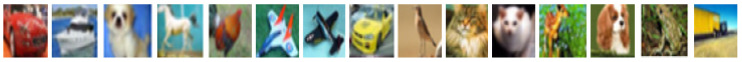
Various sample images for cifar-10 dataset [68].

This dataset [68] comprises of different semantic groups including ships, cats, cars, airplane, dogs, trucks, horses birds, deer, and frogs. This dataset contains 6000 images in every category. The presented technique provides high AP ratios for most of Cifar-10 classes. The sample images of various categories for cifar-10 database is shown in Figure 2.

The images are classified accurately due to CNN feature applied in presented approach. The Gaussian filters and non-maximal suppressions (NMS) with deep learning features make possible to efficiently classify the images from a huge range of image semantic groups—such as ships, trucks, cars, dogs, horse, frogs, deer, airplane and birds. The presented approach is expected image retrieval results with maximum throughput. At this stage, the computational load is very important issue. The presented technique is adjusted it using proper image channeling, isotroping, LOG filtering with derivations, NMS, multilevel filtering, scaling, and feature reduction in different stages; firstly, image channeling in Section 3.1 to convert color image into gray level. Secondly, isotroping and LOG filtering with derivations are applied in Section 3.2. Thirdly, applying first ordering, Kernel approximation, and recursive filtering in Section 3.4. Finally, applying PCA in Co-variants selection for detection in Section 3.9.

**Figure 3:**
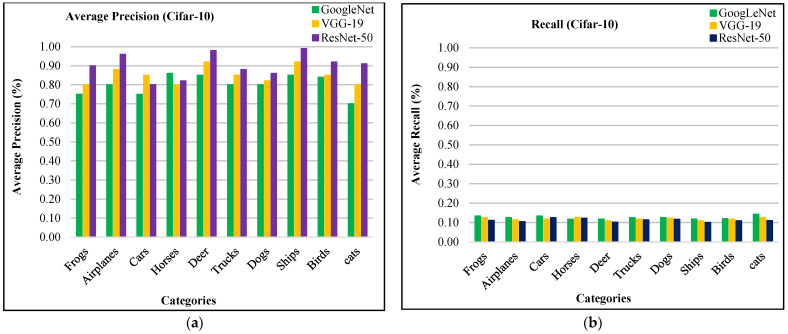
(**a**) Average precision for cifar-10 dataset; (**b**) recall for cifar-10 dataset.

In Figure 3a, the proposed method shows AP results for Cifar-10 database using ResNet-50, VGG-19, and GoogLeNet. Figure 3a reports highest AP ratios using ResNet-50 for most of the Cifar-10 categories. The presented technique shows above 85% AP rates by using features extracted from ResNet-50, above 80% AP ratios using VGG-19 and not less than 70% AP rates using GoogLeNet for most of the semantic groups of Cifar-10 dataset. The presented aooroach is outperformed for the tiny, mimicked, occupied background and multiple objected foreground images due to its object recognition capability. The image classification achieves remarkable AP ratios by presented method for cluttered, complex, and overlapping objects. The proposed approach shows significant AR results for cifar-10 in Figure 3b.

**Figure 4:**
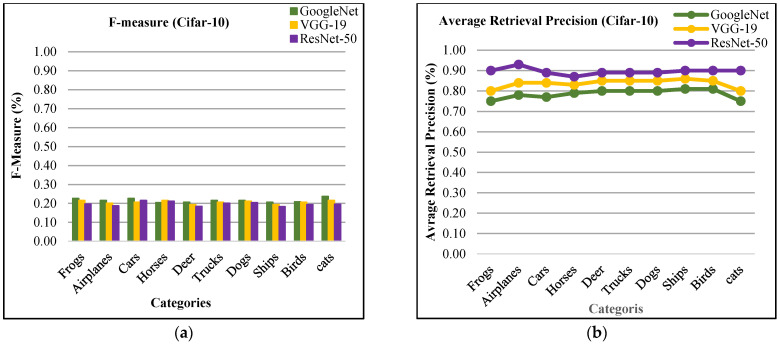
(**a**) f-measure results for cifar-10 dataset; (**b**) Average retrieval precision for cifar-10 dataset.

In Figure 4a, the presented approach reports outstanding f-measure ratios for the large, mimicked, complex foreground and background images. Moreover, the presented approach provides significant f-measure ratios using ResNet-50, VGG-19, and GoogLeNet. The proposed approach provides 90% mean average precision using ResNet-50, 85% using VGG-19, and 81% using GoogLeNet for Cifar-10. The presented approach reports outstanding ARP ratios using ResNet-50 for the categories including frogs, dogs, ships, airplanes, and birds as shown in Figure 4b. The proposed approach also shows above 85% average retrieval precision ratios for the other categories which reports significant performance for the proposed technique of Cifar-10 database. The presented approach shows better ARP rates by using features extracted from VGG-19 and GoogLeNet. 

#### 4.2.2. Performance on the Cifar-100 Dataset

**Figure 5:**
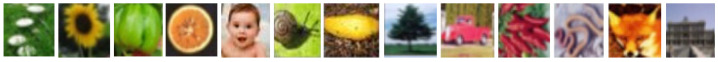
Various sample images for cifar-100 dataset [68].

This database is similar as Cifar-10 database using 32 × 32 color images rather it consists of 100 various categories. The set from [68] is comprised of different semantic groups including road, bowls, butterfly, rabbit, mountain, lamp, forest, bus, house, elephant, tractor, tiger, willow, clock, motorcycle, person, palm, rocket, etc. It contains 600 images for every category. Figure 5 shows various sample images for Cifar-100. 

Table 1 shows significant AP and f-measure ratios for Cifar-100 dataset. The presented approach reports outperformance using ResNet-50 with more than 90% AP ratio for many categories. It is noticed that proposed approach using VGG-19 shows more than 85% AP results in categories including forest, mountain, bus, rocket, butterfly, willow, tiger, person, and elephant. It is also observed that the presented approach using GoogLeNet provides 90% AP rates in motorcycle and palm, 95% in truck and 92% in road category. The strength of the presented approach provides outstanding AP results of Cifar-100.

**Figure 6:**
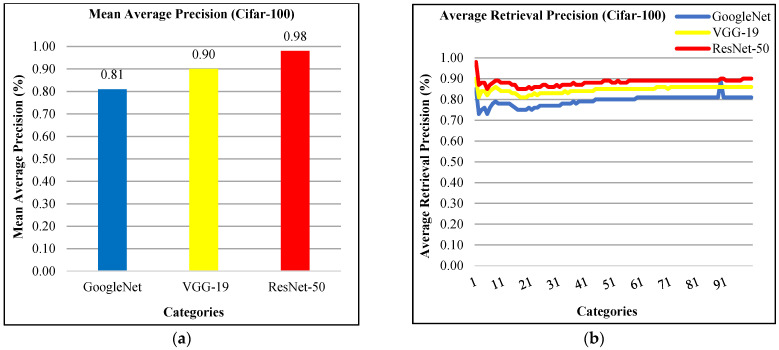
(**a**) Mean average precision for cifar-100 database; (**b**) Average retrieval precision for cifar-100 dataset.

In Figure 6a, the presented approach reports 98% mean average precision using ResNet-50, 90% using VGG-19, and 81% using GoogLeNet for Cifar-100 dataset. The presented technique provides the highest average retrieval precision ratios by using features extraction from ResNet-50 for most of the image categories which reports the significant performance of presented approach for Cifar-100 database as reported in Figure 6b. The presented method shows above-80% ARP rates using VGG-19 and better ARP rates using GoogLeNet.

#### 4.2.3. Performance on the Oxford Building Dataset

**Figure 7:**
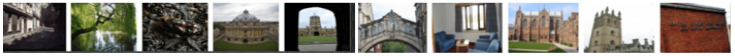
Various sample images of oxford buildings dataset [70].

This dataset [70] contains 5062 images composed from the Flickr to search for specific Oxford landmarks. The collection of images is manually classified into 11 various landmarks images, and the query set comprises of 55 images. The oxford buildings dataset is challenging for detection of cluttered and occlusion background objects. In Figure 7, the sample images of oxford buildings dataset. 

The presented approach provides significant performance using ResNet-50 with more than 85% AP rates for most of the categories as shown in Table 2. It is noticed that the presented approach using VGG-19 shows more than 80% AP ratios in categories including ashmolean, balliol, chirst church, hertford, jesus, magdalen, oriel, oxford, pitt rivers, Radcliffe, and trinity. It is also observed that the presented technique using GoogLeNet provides remarkable AP rates for most of the image categories. Moreover, the strength of the presented approach presents outstanding AP results for oxford buildings dataset. The presented approach extracts features based on shape and color which reports results accuracy. The proposed approach reports outstanding AR rates for most of the categories of Oxford buildings. Moreover, Table 2 shows f-measure results for oxford buildings database. The presented approach shows f-measure between 19% to 30% for all categories using ResNet-50, VGG-19, and GoogLeNet. In Table 2, the presented approach reports highest ARP rates using ResNet-50 for most of the categories which reports the significant performance of the proposed approach for oxford buildings dataset. The presented approach shows above 80% ARP rates using ReNet-50, 75% ARP rates using features extracted from VGG-19, and 70% ARP rates using GoogLeNet. The presented approach reports 82% mAP using ResNet-50, 90% using VGG-19, and 78% using GoogLeNet for Oxford buildings dataset.

#### 4.2.4. Performance on the ALOT (250) Dataset

**Figure 8:**
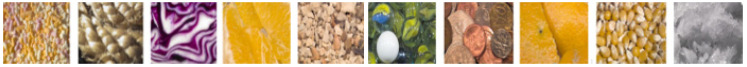
Various sample images for ALOT (250) [63].

The ALOT (250) [63] dataset is a challenging benchmark for the image classification and categorization. The ALOT (250) dataset is specially used for texture image classification. Furthermore, all classes are essential aspect for content-based image retrieval. Due to this challenge, the presented approach is experimented on a huge database contains 250 classes to test the versatility and efficiency of the presented technique. The ALOT dataset [63] comprises 250 classes by 100 samples to each. The ALOT database images have 384 × 235 pixels resolution [63]. Semantically different groups of ALOT includes clothes, spices, leaves, cigarettes, sands, vegetables, fruit, stones, sea shells, fabrics, coins and seeds, embossed fabrics, horizontal and vertical lines, bubbles, small repeated patterns, etc. These various classes are contributed for several texture information, object shapes, spatial information and objects to effectively images classification. The proposed technique efficiently classifies texture images from semantic same groups with same large, complex, overlay, texture, background and foreground objects. Gaussian filters, multi-scale filters, color coefficients, and L2 normalization steps are used by the presented model to achieve significant results for images with various textures. The images are efficiently classified by CNN features with partial derivatives, Eigenvalues, and LoG in the presented t approach. Multi-scale filtering on various levels and scale spacing are applied to achieve outstanding average precision ratios for different texture images. Most of the categories of ALOT dataset contain texture images with same colors and patterns where other categories consist of various object patterns. In Figure 8, the various sample images of ALOT (250) database are shown.

Table 3 shows remarkable AP and f-measure rates for ALOT (250) database. The proposed approach shows significant AP results up to 80% using ResNet-50 for most of the ALOT categories. It is observed that the presented approach shows better results for many image categories with various shape and color. Multiscale filtering, spatial mapping, scale spacing, and RGB coefficients with CNN features make it possible to efficiently and effectively classify images. Different images with various categories including fruits, spices, vegetables, and seeds with various color are used. The presented technique shows more than 90% AP rates using ResNet-50, above 85% using VGG-19 and above 80% using GoogLeNet for these kinds of image classes. Also, image semantic groups for bubble textures, horizontal and vertical lines, small repeated patterns, embossed fabrics, and others are classified and experimented accurately. It is also observed that presented technique shows f-measure ratios between 18% to 27% for all categories in Table 3. Moreover, the presented approach reports highest mean average precision using ResNet-50 for ALOT 250 dataset. Overall, mean average precision using ResNet-50 is 89%, mAP by using features extracted from VGG-19 is 84% and mAP using GoogLeNet is 78% for all categories of ALOT (250) database. Moreover, the presented method provides above 85% ARP rates using ResNet-50, above 80% using VGG-19, and above 70% ARP rates by using features extracted from GoogLeNet for most of the image categories of ALOT (250) database.

#### 4.2.5. Performance on the Fashion (15) Dataset

**Figure 9:**
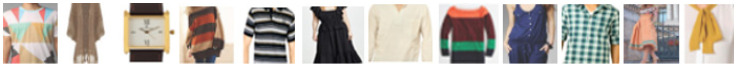
Various sample images for fashion (15) database [69].

The robustness and versatility of the presented method is experimented by testing fashion (15) database. The fashion (15) database is appropriate for analyzing texture, also it contains images with different color, texture, size, and shapes. The object classes comprise with various fabrics kinds including jersey t-shirt, undergarments, shirt, long dress, robe, blouses, coat jacket, sweater, uniform, cloak, suit, polo-sport shirt, and vest-waistcoat [69]. The fashion database contains above 260 thousand images with various complex, cluttered, background and foreground textures. Figure 9 shows sample images of fashion dataset.

**Figure 10:**
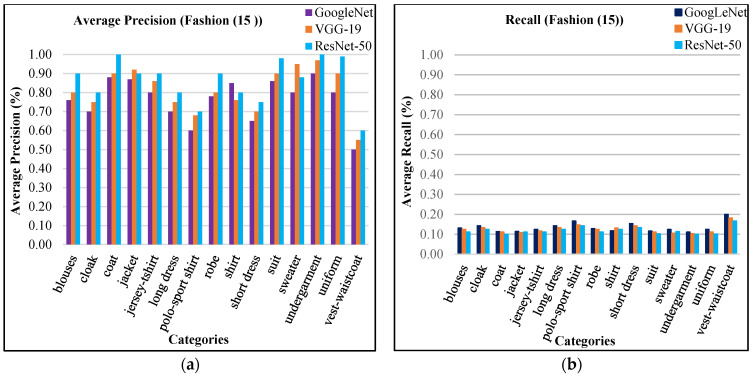
(**a**) Average precision for fashion (15) database; (**b**) Recall for fashion (15) database.

The presented approach outperforms for large, cluttered, mimicked and complex occupied objects due to its capability of object recognition. The presented approach is applied significantly image classification and provides improved average precision for complex, cluttered, and overlapping objects. The presented technique reports above 85% AP results using ResNet-50, above 75% using VGG-19, and above 70% using GoogLeNet in most of the categories as shown in Figure 10a. The presented approach is reported outstanding AP results using ResNet-50 for fashion dataset. The average recall ratios are shown in Figure 10b for fashion (15) dataset.

**Figure 11:**
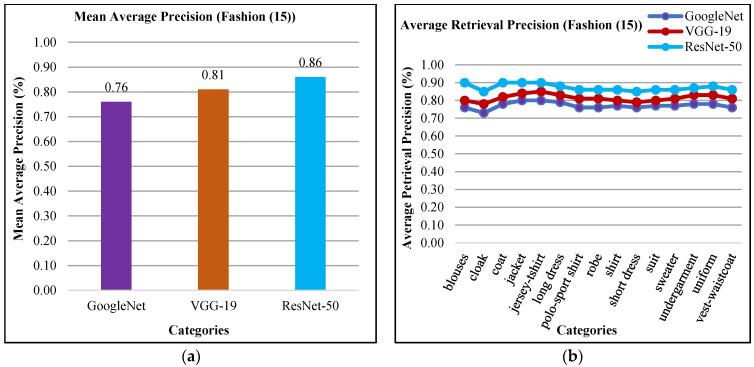
(**a**) Mean average precision of fashion (15) database; (**b**) Average retrieval precision of fashion (15) database.

The presented method shows mAP in Figure 11a of fashion dataset. The mean average precision (mAP) using ResNet-50 is 86%, mAP using VGG-19 is 81%, and mAP using GoogLeNet is 76% for all categories of fashion (15) dataset. Figure 11b shows above 85% results by using features extraction from ResNet-50, above 75% ARP rates using VGG-19 and above 70% ARP rates using GoogLeNet for many categories. The presented technique shows remarkable ARP ratios of fashion (15) database using convolutional Laplacian scaled object features with mapped colored channels to obtain the highest image retrieval rates to efficiently classify and index images. Many categories of the fashion dataset—such as jersey-t-shirt, coat, blouses, robe, uniform jacket, and long dress texture—show encouraging performance of the presented technique. Moreover, the presented method reports improved ratios between 18% to 29% for all categories using ResNet-50, VGG-19, and GoogLeNet.

#### 4.2.6. Performance on the Corel-1000 Dataset

**Figure 12:**
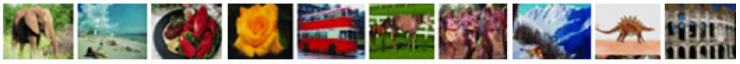
Various sample images for Corel-1000 database [38].

This database is normally applied to retrieving and classifying images [75,76,77]. The Corel-1000 dataset consists of several image classes comprising plain foreground and background images for complex and cluttered objects. This dataset consists of different semantic groups including buildings, natural scenes, mountains, people food, buses, animals, and flowers. Figure 12 shows various sample images of Corel-1000.

**Figure 13:**
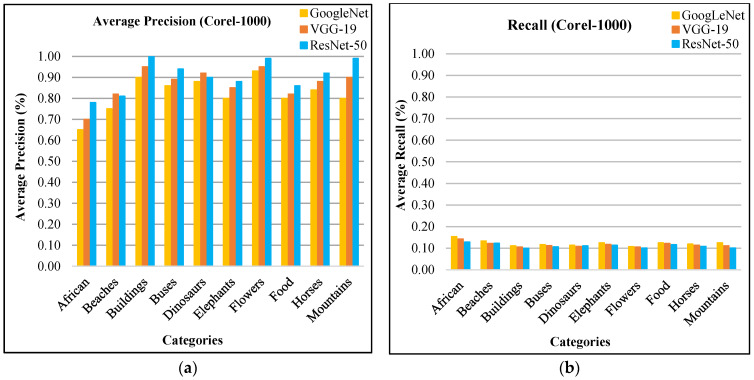
(**a**) Average precision of Corel-1000 database; (**b**) Recall of Corel-1000 database.

The AP ratios are shown in Figure 13a for Corel-1000 database. The presented approach efficiently and effectively classifies images from various kinds of groups comprising different occupied background, blobs, complex and foreground objects. The spatial mappings with CNN features, image scaling, integration, and multilevel filtering techniques make it possible to proficiently image classification. The AP rates of Corel-1000 database show dominant performance of presented approach for most of the categories due to its L2 norm, scale spacing, RGB coefficient and spatial mappings. The presented approach shows highest performance in many classes such as horse, flowers, buses, dinosaurs, buildings, food, and mountains. For complex foreground and background categories—including mountains, horses, flowers, and buildings—the presented approach reports above 90% AP ratios using ResNet-50. The category buildings and mountains show 100% and 99% AP rates using ResNet-50 respectively. Moreover, all other categories also report more than 77% AP ratios. The presented approach shows AR rates for all categories of Corel-1000 dataset in Figure 13b.

**Figure 14:**
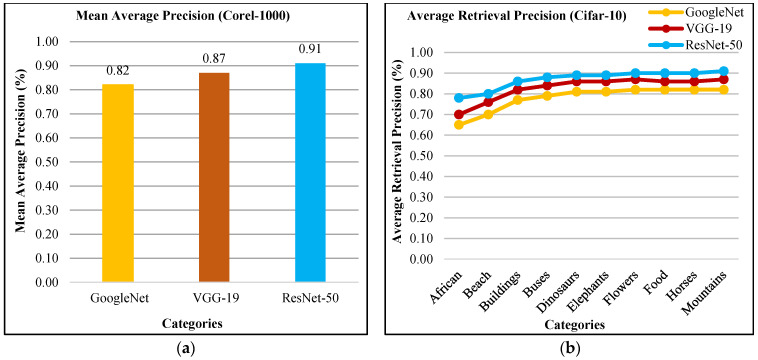
(**a**) Mean average precision of Corel-1000 database; (**b**) Average retrieval precision for Corel-1000 database.

The presented approach shows the mAP in Figure 14a for Corel-1000 dataset. The presented technique reports 91% mAP using ResNet-50, 87% using VGG-19, and 82% using GoogLeNet. In Figure 14b, the presented method shows above 87% ARP using ResNet-50, above 82% ARP rates using VGG-19, and above 75% ARP rates using GoogLeNet for many categories. The presented approach shows outstanding ARP ratios for Corel-1000 dataset using L2 norm, color coefficient, and filtering to effectively and efficiently image classification and indexing. Moreover, the presented approach shows outstanding f-measure ratios using ResNet-50, GoogLeNet, and VGG-19 for Corel-1000 database.

#### 4.2.7. Performance on the Corel-10000 Dataset

**Figure 15:**
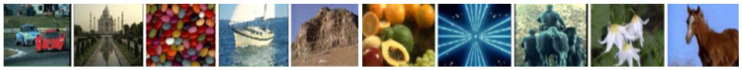
Various sample images for corel-10000 dataset [38].

The corel-10000 database [38] comprises several image classes. The corel-10000 dataset is comprised of 100 classes where each class consists of 100 images. This dataset comprises different semantic groups—including cars, flowers, texture, shining stars, butterfly, human texture flags, trees planets, ketch, hospital, text, sunset, food, animals, etc. Sample images from this dataset are shown in Figure 15.

**Figure 16:**
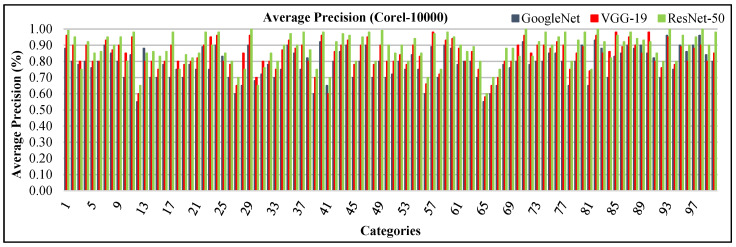
Average precision of corel-10000 database.

The presented approach provides significant AP ratios in Figure 16 for corel-10000 dataset. The AP ratio is between 60% and 100%. The presented method reports highest AP results using ResNet-50 with more than 85% AP rates for mostly classes. It is noted that the proposed method using VGG-19 shows above 80% AP results in many categories. It is also noticed that the presented technique using GoogLeNet provides above AP rates 75% in most of the categories. This dataset effectively classifies images with its deep learning feature of the presented technique. The spatial mapping and multi-scale filtering with CNN features make possible to effectively and proficiently image classification. The strength of the presented method is presented outstanding AP rates of corel-10000. The presented method provides better recall rates for all categories of corel-10000.

**Figure 17:**
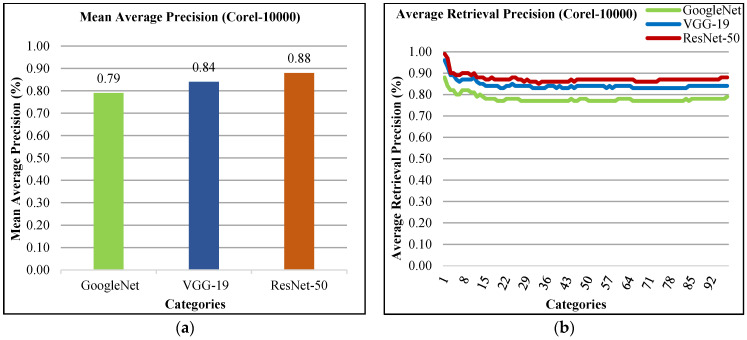
(**a**) Mean average precision for the corel-10000 database; (**b**) Average retrieval precision for the corel-10000 database.

The presented approach reports mAP results in Figure 17a. The mAP precision using ResNet-50 is 88%, mAP using VGG-19 is 84% and mAP using GoogLeNet is 79% for all categories of Corel-10000 dataset. Moreover, the presented method shows more than 85% using ResNet-50, above 80% ARP rates using VGG-19 and above 75% ARP rates using GoogLeNet for most of the categories in Figure 17b. The presented approach shows significant ARP ratios of Corel-10000 dataset using L2 normalization, RGB color coefficients to efficiently and effectively image classification and indexing. 

#### 4.2.8. Performance on the 17-Flowers Dataset

**Figure 18:**
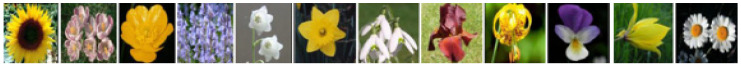
Various sample images for 17-flowers dataset [63].

This dataset [63] contains 80 images for each class. These flowers images select from very common flowers that are existed in UK. The image has attributes like light and pose variations. Some categories of flowers are different in shape but same in color. The samples images of 17-flowers dataset are shown in Figure 18. 17-flowers dataset consists various types of flowers are Lily Valley, Daffodils, Crocus, Snowdrop, Iris, Dandelion, Tulip, Bluebell, Pansy, Buttercup, Sunflower, Tigerlily, Windflower, Daisy, Colts’ Foot, Fritillary, and Cowslip.

**Figure 19:**
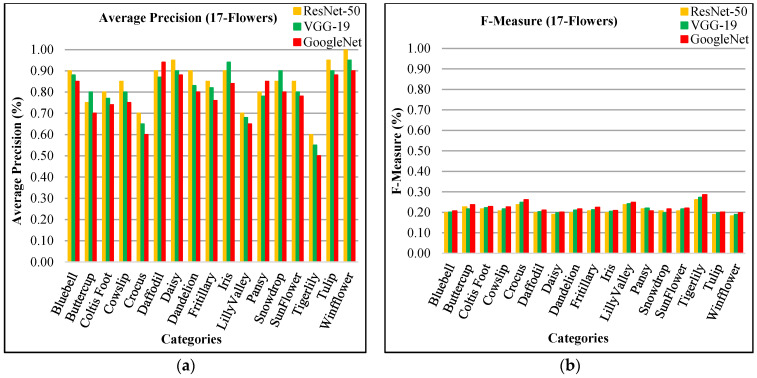
(**a**) Average precision for 17-flowers dataset; (**b**) F-measure results for 17-flowers dataset.

The presented approach shows remarkable AP rates for 17-flowers dataset as shown in Figure 19a. It is observed that the presented technique reports better results for most images of 17-flowers classes with various color, shape, and texture. The scale spacing, spatial mapping, L2 norm, and RGB coefficients with CNN features make it possible to affectively classify images of flowers. The presented technique reports significant average precision ratios using ResNet-50 in flower categories including Bluebell, Cowslip, Colts’ Foot, Daisy, Crocus, Dandelion, Tiger lily, Tulip, Fritillary, Sunflower, Lily valley, and Windflower. The versatility and superiority of the presented approach is to differentiate object depended on their color and texture which plays main role in flowers classification. Furthermore, the presented approach shows amazingly highest rates using ResNet-50 in 12/17 flower classes. The proposed technique reports high AP ratios using VGG-19 in Buttercup, Iris, and Snowdrop flower categories. It is noticed that the presented approach shows high AP ratio using GoogLeNet in Daffodils and Pansy flower categories. Sunflower and fritillary categories are similar in size and shape with different color. In Figure 19b, the presented approach shows f-measure results for 17-flowers. The proposed method shows f-measures between 18% and 30% for all image categories of 17-flowers dataset.

**Figure 20:**
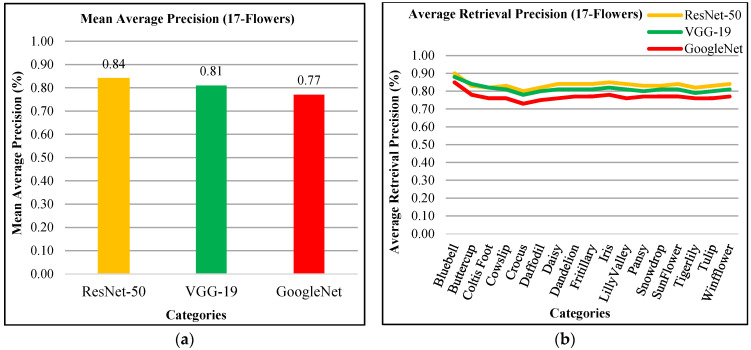
(**a**) Mean average precision for 17-flowesr dataset; (**b**) Average retrieval precision for 17-flowers dataset.

The presented approach shows 84% mAP using ResNet-50, 81% using VGG-19, and 77% using GoogLeNet for 17-flowers dataset in Figure 20a. In Figure 20b, the presented approach reports highest ARP rates using ResNet-50 for most of the categories which reports the significant performance of the proposed method for 17-flowers dataset. The presented approach presents above 80% ARP rates by using features extracted from ResNet-50, above 75% ARP using VGG-19, and 70% ARP rates using GoogLeNet.

#### 4.2.9. Performance on the FTVL Tropical Fruits Dataset

**Figure 21:**
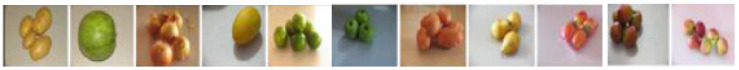
Various sample images from FTVL Tropical Fruits dataset [63].

The FTVL tropical fruits database [63] consists 2612 images of 15 various types of fruits including Tahiti Lime, Fuji Apple, Cashew, Diamond Peach, Granny Smith Apple, Asterix Potato, Nectarine, Watermelon, Honeydew Melon, Agata Potato, Spanish Pear, Plum, Kiwi, Onion, and Orange. Sample images for FTVL Tropical Fruits dataset are shown in Figure 21.

In Table 4, the presented approach shows remarkable AP, f-measure and ARP rates of the FTVL tropical fruits database. It is noted that presented technique shows highest results for most of the images of FTVL tropical fruits categories with similar shape and color. The spatial mapping, L2 norm, color coefficients, and multilevel scaling with CNN features make it possible to effectively classify images of tropical fruits. The presented technique reports high precision rates using ResNet-50 in tropical fruits categories including Tahiti Lime, Cashew, Agata Potato, Diamond Peach, Astrix Potato, Nectarine, Fuji Apple, Watermelon, Plum, Onion, and Orange. The superiority of the presented technique is to differentiate objects that are based on color, shape and texture which plays important role for classification of tropical fruits. However, the presented method shows amazingly high rates using ResNet-50 in 12/15 tropical fruits classes. The proposed approach reports highest AP rate using VGG-19 in Spanish Pear and Granny Smith Apple categories of tropical fruits. It is observed that the presented approach shows significant AP ratio using GoogLeNet in Honeydew Melon flower category. The presented approach shows outstanding f-measure results in Table 4 of FTVL Tropical Fruits database. However, the presented approach provides outstanding f-measure results using ResNet-50, VGG-19 and GoogLeNet. Moreover, the presented approach reports highest ARP rates using ResNet-50 for most of the categories which reports significant performance of the proposed method for tropical fruits dataset. The presented method presents above 90% ARP rates by using features extracted from ResNet-50, above 85% ARP using VGG-19 and 80% ARP rates using GoogLeNet. The proposed approach shows 92% mAP using ResNet-50, 88% using VGG-19, and 82% using GoogLeNet for tropical fruits dataset.

#### 4.2.10. Performance on the Caltech-256 Dataset

**Figure 22:**
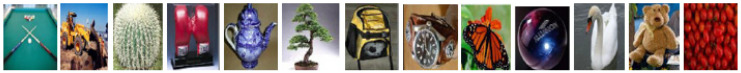
Various sample images of Caltech-256 dataset [71].

The Caltech-256 database [71] comprises of more than thirty thousand images which are allocated to 257 varied categories. Fifteen different image categories are used to perform experimentation including bonsai, wrist watch, back-pack, teddy-bear, cactus, airplane, boxing gloves, teapot, spider, billiards, swan, tomato, bulldozer, tree, and butterfly. All image categories in database are essential due to their texture patterns and background and foreground objects. Sample images of Caltech-256 dataset are shown in Figure 22.

**Figure 23:**
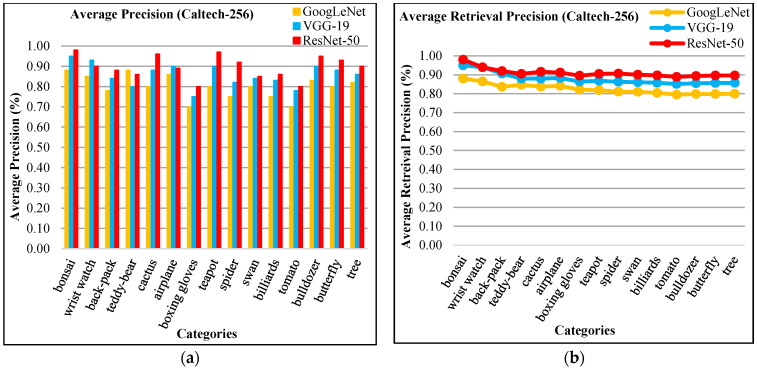
(**a**) Average precision for Caltech-256 dataset; (**b**) Average retrieval precision of Caltech-256 database.

The AP rates of Caltech-256 database is shown in Figure 23a. The presented approach provides the highest results for most of the images of Caltech-256 categories with similar shape and color. The spatial mapping, L2 norm, RGB coefficients, and scale spacing with CNN features make it possible to effectively classify images of Caltech-256. It is noted that presented method reports high precision rates using ResNet-50 in tropical fruits for most of the categories. The superiority of the presented technique is to differentiate objects that are based on color, shape, and texture which plays important role for classification of Caltech-256. However, the presented approach shows amazingly high rates using ResNet-50 in 13/15 Caltech-256. The proposed approach reports highest AP ratios using VGG-19 in wrist watch and airplane categories of Caltech-256. It is observed that the presented technique shows significant AP ratio using GoogLeNet in teddy bear category. The presented approach provides outstanding recall rates for all categories of Caltech-256. Moreover, the presented approach reports 90% mAP using ResNet-50, 86% using VGG-19, and 80% using GoogLeNet. In Figure 23b, the presented method shows above 89% ARP using ResNet-50, above 82% ARP rates using VGG-19, and above 75% ARP rates using GoogLeNet for many categories. The presented approach shows outstanding ARP ratios for Caltech-256 dataset using L2 norm, color coefficient, and multi-scale filtering to effectively and efficiently image classification and indexing.

#### 4.2.11. Results of the FTVL Tropical Fruits, Cifar-10, Corel-1000, and 17-Flowers Datasets with State-of-the-Art Methods

To check the accuracy and efficiency of the presented method, it compares with state-of-the-art methods. The comparison is performed with existing methods provided outstanding performance. 

**Figure 24:**
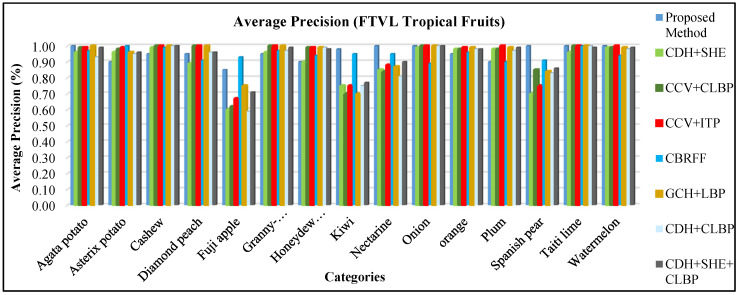
AP results of presented approach versus existing research methods for FTVL tropical fruits dataset.

The FTVL fruit dataset [63] is used for experimentations because of its cropped object, illumination differences, pose variations, and partial occlusions; and Figure 24 depicts AP rates for FTVL tropical fruits dataset, the proposed method is compared with existing methods including CBRFF [63] and with existing research techniques in [78]. The presented method shows significant average precision results in most of the image categories of FTVL tropical fruits dataset.

The AP ratios of the proposed approach technique in assessment with state-of-the-art methods are presented in Table 5. Some techniques CDH +SEH illustrate low accuracy rate due to the missing nature for their technique in the cropped objects. Some methods incorporate the textural attribute and provide AP but with mixed, ambiguous images. However, the objects with the same color and shape are still hard to recognize for them. The presented method is takes color coordinates into consideration using shape and textural properties to report 0.96 mAP.

**Figure 25:**
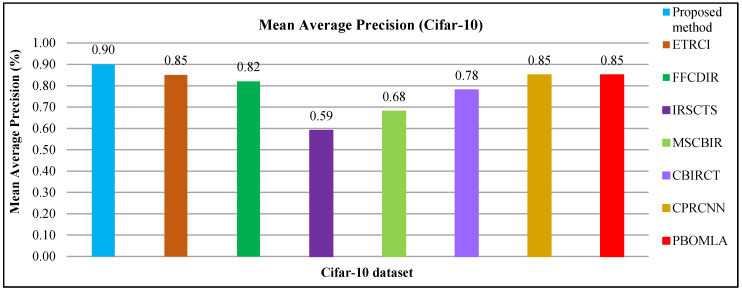
AP results of presented approach versus existing research methods for Cifar-10 dataset.

The mean average precision results are graphically shown in comparison with existing research methods for cifar-10 dataset of the presented approach and other existing research approaches are shown in Figure 25. The proposed approach shows remarkable mAP results over state of the art methods for cifar-10 dataset. The existing methods including ETRCI [76], IRSCTS [79], FFCDIR [80], MSCBIR [81], CBIRCT [82], SPRCNN [83], and PBOMLA [84]. The mean average precision of the presented approach as compared with existing research methods is graphically presented in Figure 25. 

17-Flowers database is used for shape, texture and color features experimentation. The comparative results of the proposed approach with existing research methods for mean average precision metric are shown in Table 6. The fine-grained [85] technique provides improved ratios by extracting deeper shape and color information. The research method in [86] performs the spatial matching and calculates the differences depending upon the algorithmic criteria and results in low precision due to colors and shapes. The other existing research methods [86,87,88] include linear coding and return mean average precision results. This approach is not capable to perform deep analysis on shape and texture features. The proposed approach returns outstanding AP in most of the flower categories using spatial texture and color patterns along with shape details. The mean average precision results for the proposed approach versus state-of-the-art approaches in Table 6.

**Figure 26:**
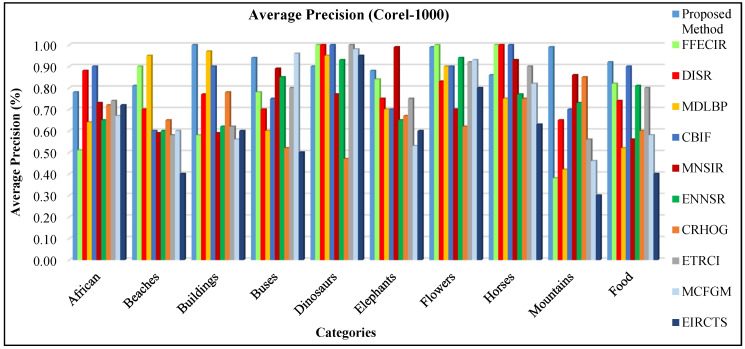
AP results of presented approach versus existing research methods for Corel-1000 dataset.

Experimentation is performed to check the accuracy and effectiveness of the presented approach, average precision results for Corel-1000 dataset are shown in comparison with state-of-the-art research approaches. The challenging research methods used for the comparison are ETRCI [76], DISR [73], CBIF [63], FFECIR [91], MDLBP [75], MNSIR [81], ENNSR [92], CRHOG [93], MCFGM [94], and EIRCTS [79]. In Figure 26, a graphical illustration of AP rates for the presented approach as compared with state-of-the-art research methods. The presented technique shows the highest AP results performance in most of the image categories of Corel-1000 dataset. The proposed approach reports better AP rates in the categories—such as buildings, flowers, mountains, and food. 

**Figure 27:**
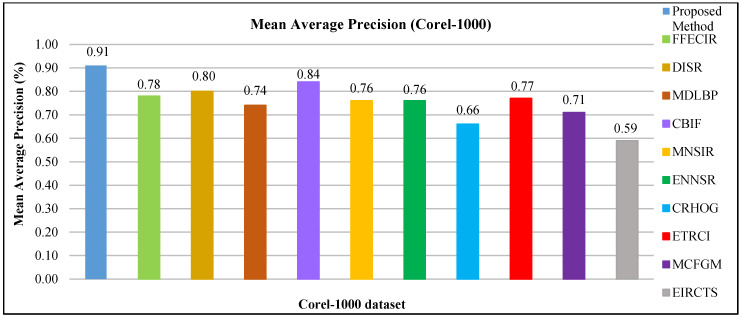
mAP results of presented approach versus existing research methods for Corel-1000 dataset.

Figure 27 shows mAP results of the presented approach in comparison with state-of-the-art research methods. The proposed approach reports highest 0.91 mAP. CBIF [63] shows second highest 0.84 mAP. MNSIR [81], ENNSR [92] report 0.76 mAP. ETRCI [76], DISR [73], FFECIR [91], MDLBP [75], CRHOG [93], and MCFGM [94] show mean average precision ratios between 0.66 and 0.80. EIRCTS [79] provides the lowest 0.59 mAP. The EIRCTS [75] covers the textural aspects therefore mAP rates are comparatively lower than the other approaches.

## 5. Conclusions

This research presents a novel interactional fusion of GoogLeNet, VGG-19, and ResNet-50 with an innovative salient anchor collection and detection framework to enhance the image retrieval accuracy over large datasets. This innovative deep learning solution is a breakthrough in framework capsulation, features binding, primitive features fusion with layered technology, deep features orientation, and CNN typed effects on revealed local and global signatures. The remarkable outcomes with extensive experimentation from benchmark architectures endorsed the superiority of the presented approach with ALOT (250), Corel-10000, Cifar-10, Oxford Buildings, FTVL Tropical Fruits, 17-Flowers, Cifar-100, Fashion (15), Corel-1000, and Caltech-256 highly recognizable datasets. An extension to this research is the fusion of several formulations.

## Figures and Tables

**Table 1:** Average precision and f-measure ratios of Cifar-100 dataset.

Cifar-100 Dataset (Average Precision and F-Measure)													
Category	ResNet-50		VGG-19		GoogLeNet		Category	ResNet-50		VGG-19		GoogLeNet	
1	0.98	0.18	0.90	0.20	0.85	0.21	51	0.85	0.21	0.80	0.22	0.75	0.23
2	0.75	0.23	0.72	0.23	0.60	0.26	52	0.96	0.19	0.92	0.19	0.85	0.21
3	0.92	0.19	0.96	0.20	0.80	0.22	53	0.80	0.22	0.85	0.23	0.70	0.24
4	0.86	0.20	0.83	0.21	0.80	0.22	54	0.94	0.19	0.90	0.20	0.84	0.21
5	0.72	0.23	0.75	0.23	0.60	0.26	55	0.85	0.21	0.83	0.21	0.75	0.23
6	0.98	0.18	0.96	0.19	0.90	0.20	56	0.98	0.18	0.95	0.19	0.90	0.20
7	0.97	0.19	0.90	0.20	0.88	0.20	57	0.97	0.19	0.94	0.19	0.89	0.20
8	0.92	0.19	0.88	0.20	0.85	0.21	58	0.90	0.20	0.87	0.20	0.85	0.21
9	0.88	0.20	0.80	0.22	0.90	0.22	59	0.88	0.20	0.85	0.21	0.90	0.20
10	0.80	0.22	0.75	0.23	0.70	0.24	60	0.95	0.19	0.90	0.20	0.88	0.20
11	0.86	0.20	0.88	0.20	0.80	0.22	61	0.82	0.21	0.88	0.20	0.78	0.22
12	0.95	0.19	0.86	0.20	0.80	0.22	62	0.86	0.20	0.80	0.22	0.82	0.21
13	0.90	0.20	0.84	0.21	0.78	0.22	63	0.89	0.20	0.90	0.20	0.80	0.22
14	0.70	0.24	0.63	0.25	0.61	0.26	64	0.90	0.20	0.87	0.20	0.81	0.21
15	0.83	0.21	0.78	0.22	0.70	0.24	65	0.93	0.19	0.90	0.20	0.86	0.20
16	0.65	0.25	0.70	0.24	0.60	0.26	66	0.92	0.19	0.88	0.20	0.85	0.21
17	0.70	0.24	0.75	0.23	0.70	0.24	67	0.95	0.19	0.90	0.20	0.88	0.20
18	0.88	0.20	0.80	0.22	0.80	0.22	68	0.88	0.20	0.86	0.20	0.90	0.21
19	0.90	0.20	0.93	0.21	0.70	0.24	69	0.88	0.20	0.84	0.21	0.80	0.22
20	0.96	0.19	0.95	0.19	0.89	0.20	70	0.83	0.21	0.80	0.22	0.70	0.24
21	0.83	0.21	0.78	0.22	0.70	0.24	71	1.00	0.18	0.95	0.19	0.92	0.19
22	0.98	0.18	0.96	0.19	0.90	0.20	72	0.83	0.21	0.81	0.21	0.80	0.22
23	0.83	0.21	0.81	0.21	0.85	0.21	73	0.92	0.19	0.90	0.20	0.80	0.22
24	0.98	0.18	0.95	0.19	0.90	0.20	74	0.98	0.18	0.90	0.20	0.80	0.22
25	0.92	0.19	0.94	0.19	0.83	0.21	75	0.90	0.20	0.87	0.20	0.80	0.22
26	0.85	0.21	0.80	0.22	0.80	0.22	76	0.95	0.19	0.97	0.20	0.80	0.22
27	0.80	0.22	0.78	0.22	0.70	0.24	77	0.98	0.18	0.95	0.19	0.90	0.20
28	0.75	0.23	0.70	0.24	0.60	0.26	78	0.80	0.22	0.70	0.24	0.65	0.25
29	1.00	0.18	0.95	0.19	0.90	0.20	79	0.93	0.19	0.97	0.19	0.90	0.20
30	0.99	0.18	0.92	0.19	0.88	0.20	80	0.98	0.18	0.94	0.19	0.89	0.20
31	0.76	0.22	0.80	0.22	0.72	0.23	81	0.75	0.23	0.70	0.24	0.60	0.26
32	0.96	0.19	0.88	0.20	0.92	0.19	82	1.00	0.18	0.98	0.18	0.93	0.19
33	0.94	0.19	0.90	0.20	0.95	0.19	83	0.92	0.19	0.90	0.20	0.88	0.20
34	0.85	0.21	0.80	0.22	0.75	0.23	84	0.82	0.21	0.75	0.23	0.65	0.25
35	0.97	0.19	0.90	0.20	0.86	0.20	85	0.96	0.19	0.88	0.20	0.82	0.21
36	1.00	0.18	0.94	0.19	0.88	0.20	86	0.92	0.19	0.80	0.22	0.88	0.20
37	0.80	0.22	0.82	0.21	0.75	0.23	87	0.98	0.18	0.95	0.19	0.90	0.20
38	0.87	0.20	0.85	0.21	0.80	0.22	88	0.94	0.19	0.90	0.20	0.88	0.20
39	0.88	0.20	0.80	0.22	0.83	0.21	89	0.93	0.19	0.96	0.19	0.90	0.20
40	0.98	0.18	0.90	0.20	0.89	0.20	90	0.92	0.19	0.85	0.21	0.80	0.22
41	0.85	0.21	0.88	0.20	0.84	0.21	91	0.85	0.21	0.82	0.21	0.82	0.21
42	0.92	0.19	0.85	0.21	0.82	0.21	92	0.80	0.22	0.70	0.24	0.66	0.25
43	0.97	0.19	0.95	0.19	0.88	0.20	93	1.00	0.18	0.96	0.19	0.90	0.20
44	0.96	0.19	0.92	0.19	0.90	0.20	94	0.80	0.22	0.75	0.23	0.75	0.23
45	0.92	0.19	0.96	0.19	0.89	0.20	95	0.96	0.19	0.80	0.22	0.90	0.20
46	1.00	0.18	0.98	0.18	0.92	0.19	96	0.90	0.20	0.82	0.21	0.92	0.20
47	0.98	0.18	0.90	0.20	0.85	0.21	97	0.95	0.19	0.90	0.20	0.88	0.20
48	0.94	0.19	0.90	0.20	0.88	0.20	98	1.00	0.18	0.98	0.18	0.90	0.20
49	0.89	0.20	0.87	0.20	0.85	0.21	99	0.90	0.20	0.92	0.21	0.80	0.22
50	0.75	0.23	0.72	0.23	0.70	0.24	100	0.98	0.18	0.90	0.20	0.86	0.20

**Table 2:** Average precision, F-measure, and ARP ratios for Oxford buildings dataset.

Oxford Buildings Dataset (Average Precision, F-Measure, and ARP)									
Category	ResNet-50			VGG-19			GoogLeNet		
All soul	0.65	0.20	0.65	0.60	0.26	0.60	0.58	0.27	0.58
Ashmolean	0.88	0.21	0.77	0.85	0.21	0.73	0.80	0.22	0.69
Balliol	0.82	0.25	0.78	0.75	0.23	0.73	0.70	0.24	0.69
Bodleain	0.64	0.19	0.75	0.60	0.26	0.70	0.50	0.29	0.65
Chirst church	0.95	0.23	0.79	0.90	0.20	0.74	0.87	0.20	0.69
Corner market	0.75	0.20	0.78	0.72	0.23	0.74	0.70	0.24	0.69
Hertford	0.88	0.19	0.80	0.82	0.21	0.75	0.90	0.20	0.72
Jesus	0.96	0.22	0.82	0.90	0.20	0.77	0.88	0.20	0.74
Keble	0.76	0.18	0.81	0.75	0.23	0.77	0.70	0.24	0.74
Magdalen	1.00	0.22	0.83	0.97	0.19	0.79	0.90	0.20	0.75
New	0.80	0.20	0.83	0.75	0.23	0.78	0.65	0.25	0.74
Oriel	0.86	0.21	0.83	0.90	0.20	0.79	0.83	0.21	0.75
Oxford	0.85	0.19	0.83	0.88	0.20	0.80	0.86	0.20	0.76
Pitt rivers	0.94	0.20	0.84	0.94	0.19	0.91	0.90	0.20	0.77
Radcliffe	0.90	0.18	0.84	0.95	0.19	0.82	0.88	0.20	0.78
Trinity	0.98	0.22	0.85	0.92	0.19	0.83	0.84	0.21	0.78
Worcester	0.80	0.20	0.85	0.78	0.22	0.82	0.70	0.24	0.78

**Table 3:** Average precision and F-measure ratios for ALOT (250) dataset.

ALOT (250) Dataset (Average Precision and F-Measure)													
**Bubble Textures**													
**Category**	**ResNet-50**		**VGG-19**		**GoogLeNet**		**Category**	**ResNet-50**		**VGG-19**		**GoogLeNet**	
1	0.80	0.19	0.90	0.20	0.85	0.21	12	0.92	0.18	0.90	0.19	0.80	0.20
2	0.94	0.22	0.75	0.23	0.72	0.23	13	0.98	0.20	0.96	0.20	0.90	0.21
3	0.85	0.18	0.90	0.20	0.82	0.21	14	0.91	0.22	0.88	0.24	0.83	0.25
4	0.98	0.19	0.95	0.19	0.90	0.20	15	0.80	0.20	0.70	0.19	0.65	0.20
5	0.97	0.19	0.88	0.20	0.80	0.22	16	0.90	0.22	0.92	0.24	0.90	0.26
6	0.90	0.21	0.86	0.20	0.84	0.21	17	0.76	0.20	0.70	0.20	0.60	0.22
7	0.88	0.20	0.82	0.21	0.70	0.24	18	0.88	0.22	0.86	0.22	0.80	0.24
8	0.95	0.19	0.90	0.20	0.80	0.22	19	0.80	0.21	0.76	0.22	0.70	0.22
9	0.82	0.19	0.89	0.20	0.80	0.22	20	0.85	0.20	0.80	0.21	0.78	0.22
10	0.86	0.19	0.90	0.20	0.78	0.22	21	0.89	0.18	0.82	0.19	0.80	0.20
11	0.89	0.24	0.60	0.26	0.55	0.27							
**Stone Textures**													
**Category**	**ResNet-50**		**VGG-19**		**GoogLeNet**		**Category**	**ResNet-50**		**VGG-19**		**GoogLeNet**	
1	0.70	0.24	0.65	0.25	0.60	0.26	14	0.92	0.19	0.88	0.20	0.80	0.22
2	0.85	0.21	0.80	0.22	0.86	0.20	15	0.98	0.18	0.93	0.19	0.90	0.20
3	0.65	0.25	0.60	0.26	0.50	0.29	16	0.83	0.21	0.80	0.22	0.85	0.21
4	0.80	0.22	0.76	0.22	0.70	0.24	17	0.96	0.19	0.92	0.19	0.90	0.20
5	0.90	0.20	0.86	0.20	0.80	0.22	18	0.88	0.20	0.86	0.20	0.82	0.21
6	0.90	0.20	0.82	0.21	0.75	0.23	19	0.92	0.19	0.87	0.20	0.80	0.22
7	0.60	0.26	0.55	0.27	0.50	0.29	20	0.83	0.21	0.75	0.23	0.70	0.24
8	0.88	0.20	0.80	0.22	0.74	0.23	21	0.85	0.21	0.80	0.22	0.78	0.22
9	0.87	0.20	0.80	0.22	0.73	0.23	22	0.86	0.20	0.83	0.21	0.80	0.22
10	0.98	0.18	0.89	0.20	0.88	0.20	23	0.90	0.20	0.92	0.19	0.80	0.22
11	0.86	0.20	0.82	0.21	0.70	0.24	24	0.86	0.20	0.80	0.22	0.77	0.22
12	0.86	0.20	0.80	0.22	0.75	0.23	25	0.80	0.22	0.76	0.22	0.72	0.23
13	0.94	0.19	0.90	0.20	0.87	0.20	26	0.88	0.20	0.83	0.21	0.80	0.22
**Leaf Textures**													
**Category**	**ResNet-50**		**VGG-19**		**GoogLeNet**		**Category**	**ResNet-50**		**VGG-19**		**GoogLeNet**	
1	0.76	0.22	0.72	0.23	0.68	0.24	15	0.76	0.22	0.72	0.23	0.70	0.24
2	0.93	0.19	0.88	0.20	0.85	0.21	16	0.80	0.22	0.78	0.22	0.70	0.24
3	0.96	0.19	0.91	0.20	0.83	0.21	17	0.91	0.20	0.88	0.20	0.80	0.22
4	0.80	0.22	0.74	0.23	0.70	0.24	18	0.88	0.20	0.80	0.22	0.76	0.22
5	0.95	0.19	0.90	0.20	0.80	0.22	19	0.90	0.20	0.87	0.20	0.84	0.21
6	0.86	0.20	0.81	0.21	0.76	0.22	20	0.87	0.20	0.82	0.21	0.80	0.22
7	0.90	0.20	0.88	0.20	0.83	0.21	21	0.86	0.20	0.80	0.22	0.79	0.22
8	0.80	0.22	0.75	0.23	0.70	0.24	22	0.80	0.22	0.70	0.24	0.63	0.25
9	0.96	0.19	0.90	0.20	0.80	0.22	23	0.90	0.20	0.88	0.20	0.86	0.20
10	0.98	0.18	0.92	0.19	0.88	0.20	24	0.91	0.20	0.80	0.22	0.78	0.22
11	0.87	0.20	0.81	0.21	0.78	0.22	25	0.96	0.19	0.90	0.20	0.80	0.22
12	0.92	0.19	0.90	0.20	0.83	0.21	26	0.98	0.18	0.88	0.20	0.83	0.21
13	0.97	0.19	0.92	0.19	0.88	0.20	27	0.89	0.20	0.80	0.22	0.76	0.22
14	0.96	0.19	0.94	0.19	0.92	0.19	28	0.80	0.22	0.78	0.22	0.75	0.23
**Fabric Textures**													
**Category**	**ResNet-50**		**VGG-19**		**GoogLeNet**		**Category**	**ResNet-50**		**VGG-19**		**GoogLeNet**	
1	0.90	0.22	0.83	0.21	0.80	0.22	19	0.95	0.19	0.92	0.19	0.82	0.21
2	0.96	0.21	0.90	0.20	0.82	0.21	20	0.98	0.18	0.95	0.19	0.90	0.20
3	0.98	0.20	0.90	0.20	0.88	0.20	21	0.80	0.22	0.75	0.23	0.70	0.24
4	0.94	0.21	0.88	0.20	0.84	0.21	22	0.75	0.23	0.70	0.24	0.60	0.26
5	0.80	0.24	0.75	0.23	0.67	0.24	23	0.90	0.20	0.88	0.20	0.88	0.20
6	0.89	0.22	0.84	0.21	0.80	0.22	24	0.98	0.18	0.90	0.20	0.82	0.21
7	0.86	0.24	0.80	0.22	0.70	0.24	25	0.95	0.19	0.90	0.20	0.88	0.20
8	0.92	0.22	0.86	0.20	0.80	0.22	26	0.90	0.20	0.88	0.20	0.81	0.21
9	0.88	0.24	0.82	0.21	0.70	0.24	27	0.98	0.18	0.95	0.19	0.90	0.20
10	0.98	0.22	0.89	0.20	0.80	0.22	28	0.94	0.19	0.90	0.20	0.80	0.22
11	0.80	0.22	0.82	0.21	0.80	0.22	29	0.96	0.19	0.92	0.19	0.88	0.20
12	0.92	0.21	0.90	0.20	0.83	0.21	30	0.86	0.20	0.80	0.22	0.74	0.23
13	0.98	0.22	0.90	0.20	0.80	0.22	31	0.88	0.20	0.85	0.21	0.80	0.22
14	0.96	0.21	0.92	0.19	0.82	0.21	32	0.92	0.19	0.90	0.20	0.80	0.22
15	0.94	0.20	0.91	0.20	0.90	0.20	33	0.98	0.18	0.92	0.19	0.82	0.21
16	0.90	0.22	0.86	0.20	0.80	0.22	34	0.97	0.19	0.92	0.19	0.85	0.21
17	0.78	0.26	0.70	0.24	0.60	0.26	35	0.94	0.19	0.90	0.20	0.80	0.22
18	0.85	0.24	0.80	0.22	0.70	0.24	36	0.89	0.20	0.83	0.21	0.76	0.22
**Vegetable Textures**													
**Category**	**ResNet-50**		**VGG-19**		**GoogLeNet**		**Category**	**ResNet-50**		**VGG-19**		**GoogLeNet**	
1	0.98	0.18	0.90	0.20	0.87	0.20	7	0.80	0.22	0.70	0.24	0.66	0.25
2	0.88	0.20	0.80	0.22	0.75	0.23	8	0.90	0.20	0.80	0.22	0.76	0.22
3	0.90	0.20	0.87	0.20	0.80	0.22	9	0.65	0.25	0.60	0.26	0.50	0.29
4	0.92	0.19	0.88	0.20	0.82	0.21	10	0.98	0.18	0.94	0.19	0.90	0.20
5	0.98	0.18	0.93	0.19	0.90	0.20	11	0.70	0.24	0.66	0.25	0.60	0.26
6	0.90	0.20	0.82	0.21	0.74	0.23	12	0.96	0.19	0.90	0.20	0.80	0.22
**Fruit Textures**													
**Category**	**ResNet-50**		**VGG-19**		**GoogLeNet**		**Category**	**ResNet-50**		**VGG-19**		**GoogLeNet**	
1	0.92	0.19	0.88	0.20	0.80	0.22	11	0.89	0.18	0.80	0.22	0.70	0.24
2	0.90	0.20	0.87	0.20	0.83	0.21	12	0.93	0.20	0.88	0.20	0.83	0.21
3	0.88	0.20	0.82	0.21	0.73	0.23	13	0.96	0.19	0.90	0.20	0.80	0.22
4	0.86	0.20	0.80	0.22	0.78	0.22	14	0.95	0.19	0.93	0.19	0.85	0.21
5	0.98	0.18	0.90	0.20	0.81	0.21	15	0.97	0.19	0.95	0.19	0.90	0.20
6	0.97	0.19	0.95	0.19	0.82	0.21	16	0.89	0.19	0.85	0.21	0.80	0.22
7	0.94	0.19	0.90	0.20	0.88	0.20	17	0.98	0.20	0.92	0.19	0.88	0.20
8	0.92	0.19	0.91	0.20	0.82	0.21	18	0.95	0.18	0.90	0.20	0.82	0.21
9	0.80	0.22	0.78	0.22	0.70	0.24	19	0.75	0.19	0.73	0.23	0.70	0.24
10	0.99	0.18	0.95	0.19	0.90	0.20	20	0.89	0.23	0.80	0.22	0.70	0.24
**Seed** **Textures**													
**Category**	**ResNet-50**		**VGG-19**		**GoogLeNet**		**Category**	**ResNet-50**		**VGG-19**		**GoogLeNet**	
1	0.78	0.22	0.75	0.23	0.70	0.24	8	0.90	0.20	0.83	0.21	0.80	0.22
2	0.99	0.18	0.94	0.19	0.90	0.20	9	0.89	0.20	0.84	0.21	0.81	0.21
3	0.96	0.19	0.92	0.19	0.88	0.20	10	0.92	0.19	0.86	0.20	0.82	0.21
4	0.92	0.19	0.88	0.20	0.82	0.21	11	0.95	0.19	0.92	0.19	0.83	0.21
5	0.88	0.20	0.84	0.21	0.80	0.22	12	0.98	0.18	0.94	0.19	0.86	0.20
6	0.95	0.19	0.90	0.20	0.83	0.21	13	0.95	0.19	0.92	0.19	0.88	0.20
7	0.80	0.22	0.76	0.22	0.70	0.24	14	0.70	0.24	0.66	0.25	0.60	0.26
**Bean Textures**													
**Category**	**ResNet-50**		**VGG-19**		**GoogLeNet**		**Category**	**ResNet-50**		**VGG-19**		**GoogLeNet**	
1	0.89	0.20	0.85	0.21	0.83	0.21	9	0.95	0.19	0.94	0.19	0.90	0.20
2	0.87	0.20	0.82	0.21	0.70	0.24	10	0.98	0.18	0.92	0.19	0.87	0.20
3	0.90	0.20	0.86	0.20	0.83	0.21	11	0.60	0.26	0.55	0.27	0.50	0.29
4	0.90	0.20	0.82	0.21	0.80	0.22	12	0.80	0.22	0.82	0.21	0.84	0.21
5	0.75	0.23	0.70	0.24	0.60	0.26	13	0.85	0.21	0.81	0.21	0.80	0.22
6	0.98	0.18	0.90	0.20	0.87	0.20	14	0.76	0.22	0.80	0.22	0.70	0.24
7	0.60	0.26	0.55	0.27	0.52	0.28	15	0.90	0.20	0.85	0.21	0.76	0.22
8	0.90	0.20	0.82	0.21	0.76	0.22							
**Coin Textures**													
**Category**	**ResNet-50**		**VGG-19**		**GoogLeNet**		**Category**	**ResNet-50**		**VGG-19**		**GoogLeNet**	
1	0.87	0.20	0.80	0.22	0.74	0.23	12	0.80	0.22	0.75	0.23	0.72	0.23
2	0.90	0.20	0.86	0.20	0.82	0.21	13	0.90	0.20	0.86	0.20	0.81	0.21
3	0.94	0.19	0.82	0.21	0.78	0.22	14	0.90	0.20	0.88	0.20	0.80	0.22
4	0.98	0.18	0.86	0.20	0.82	0.21	15	0.80	0.22	0.75	0.23	0.70	0.24
5	0.95	0.19	0.90	0.20	0.80	0.22	16	0.98	0.18	0.95	0.19	0.90	0.20
6	0.94	0.19	0.88	0.20	0.84	0.21	17	0.96	0.19	0.90	0.20	0.82	0.21
7	0.98	0.18	0.90	0.20	0.80	0.22	18	0.86	0.20	0.84	0.21	0.80	0.22
8	0.89	0.20	0.82	0.21	0.75	0.23	19	0.89	0.20	0.83	0.21	0.79	0.22
9	0.87	0.20	0.81	0.21	0.76	0.22	20	0.98	0.18	0.95	0.19	0.88	0.20
10	0.95	0.19	0.92	0.19	0.84	0.21	21	0.89	0.20	0.85	0.21	0.80	0.22
11	0.87	0.20	0.82	0.21	0.70	0.24	22	0.90	0.20	0.84	0.21	0.78	0.22
**Sea Shell Textures**													
**Category**	**ResNet-50**		**VGG-19**		**GoogLeNet**		**Category**	**ResNet-50**		**VGG-19**		**GoogLeNet**	
1	0.98	0.18	0.95	0.19	0.85	0.21	11	0.89	0.20	0.83	0.21	0.76	0.22
2	0.98	0.19	0.93	0.19	0.82	0.20	12	0.91	0.20	0.88	0.20	0.82	0.21
3	0.96	0.19	0.92	0.20	0.88	0.20	13	0.95	0.19	0.90	0.20	0.84	0.21
4	0.92	0.20	0.88	0.21	0.86	0.22	14	0.96	0.19	0.92	0.19	0.80	0.22
5	0.90	0.22	0.83	0.22	0.80	0.24	15	0.98	0.18	0.92	0.19	0.90	0.20
6	0.80	0.18	0.78	0.19	0.70	0.21	16	0.80	0.22	0.79	0.22	0.73	0.23
7	0.98	0.18	0.94	0.19	0.83	0.20	17	0.55	0.27	0.53	0.28	0.50	0.29
8	0.99	0.18	0.93	0.20	0.88	0.21	18	0.75	0.23	0.72	0.23	0.70	0.24
9	0.98	0.19	0.90	0.19	0.83	0.22	19	0.86	0.20	0.80	0.22	0.72	0.23
10	0.96	0.18	0.92	0.19	0.80	0.21	20	0.98	0.18	0.88	0.20	0.83	0.21
**Spice Textures**													
**Category**	**ResNet-50**		**VGG-19**		**GoogLeNet**		**Category**	**ResNet-50**		**VGG-19**		**GoogLeNet**	
1	0.97	0.19	0.84	0.21	0.80	0.22	19	0.95	0.19	0.91	0.20	0.86	0.20
2	0.92	0.19	0.90	0.20	0.90	0.20	20	0.98	0.18	0.94	0.19	0.88	0.20
3	0.90	0.20	0.82	0.21	0.74	0.23	21	0.89	0.20	0.85	0.21	0.80	0.22
4	0.75	0.23	0.70	0.24	0.65	0.25	22	0.85	0.21	0.80	0.22	0.70	0.24
5	0.75	0.23	0.73	0.23	0.68	0.24	23	0.98	0.18	0.90	0.20	0.81	0.21
6	0.99	0.18	0.90	0.20	0.87	0.20	24	0.94	0.19	0.92	0.19	0.82	0.21
7	0.60	0.26	0.58	0.27	0.50	0.29	25	0.98	0.18	0.95	0.19	0.90	0.20
8	0.88	0.20	0.80	0.22	0.70	0.24	26	0.88	0.20	0.80	0.22	0.76	0.22
9	0.95	0.19	0.90	0.20	0.84	0.21	27	0.86	0.20	0.82	0.21	0.70	0.24
10	0.80	0.22	0.77	0.22	0.72	0.23	28	0.98	0.18	0.90	0.20	0.83	0.21
11	0.94	0.19	0.90	0.20	0.80	0.22	29	0.94	0.19	0.88	0.20	0.80	0.22
12	0.89	0.20	0.84	0.21	0.80	0.22	30	0.92	0.19	0.84	0.21	0.80	0.22
13	0.86	0.20	0.80	0.22	0.76	0.22	31	0.97	0.19	0.94	0.19	0.82	0.21
14	0.85	0.21	0.80	0.22	0.74	0.23	32	0.88	0.20	0.80	0.22	0.70	0.24
15	0.97	0.19	0.84	0.21	0.80	0.22	33	0.89	0.20	0.84	0.21	0.78	0.22
16	0.92	0.19	0.90	0.20	0.90	0.20	34	0.96	0.19	0.90	0.20	0.76	0.22
17	0.90	0.20	0.82	0.21	0.74	0.23	35	0.99	0.18	0.96	0.19	0.90	0.20
18	0.75	0.23	0.70	0.24	0.65	0.25	36	0.90	0.20	0.88	0.20	0.85	0.21

**Table 4:** Average precision, F-measure, and ARP ratios of FTVL Tropical Fruits dataset.

FTVL Tropical Fruits Dataset (Average Precision, F-Measure, and ARP)									
Category	ResNet-50			VGG-19			GoogLeNet		
Tahiti Lime	1.00	0.18	0.90	0.86	0.20	0.86	0.80	0.22	0.80
Cashew	0.95	0.19	0.98	0.90	0.20	0.88	0.87	0.20	0.84
Agata Potato	1.00	0.18	0.98	0.92	0.19	0.89	0.90	0.20	0.86
Diamond Peach	0.95	0.19	0.98	0.89	0.20	0.89	0.82	0.21	0.85
Granny Smith Apple	0.95	0.19	0.97	0.90	0.20	0.89	0.75	0.23	0.83
Asterix Potato	0.90	0.20	0.96	0.88	0.20	0.89	0.70	0.24	0.81
Nectarine	1.00	0.18	0.96	0.90	0.20	0.89	0.86	0.20	0.81
Fuji Apple	0.85	0.21	0.95	0.78	0.22	0.88	0.70	0.24	0.80
Watermelon	1.00	0.18	0.96	0.98	0.18	0.89	0.90	0.20	0.81
Honeydew Melon	0.90	0.20	0.95	0.77	0.22	0.88	0.82	0.21	0.81
Spanish Pear	1.00	0.18	0.95	0.98	0.18	0.89	0.88	0.20	0.82
Plum	0.90	0.20	0.95	0.88	0.20	0.89	0.80	0.22	0.82
Kiwi	0.98	0.20	0.95	0.78	0.22	0.88	0.76	0.22	0.81
Onion	1.00	0.18	0.95	0.90	0.20	0.88	0.88	0.20	0.82
Orange	0.95	0.19	0.95	0.88	0.20	0.88	0.80	0.22	0.82

**Table 5:** Comparison of presented method mAP results with CBRFF [63] and other state of the art methods [78] of FTVL Tropical Fruits dataset.

FTVL Tropical Fruits Dataset Vs. Existing Methods–Mean Average Precision (mAP)								
**Average**	**Proposed Method**	**CDH + ** **SEH**	**CCV + ** **CLBP**	**CCV + ** **LTP**	**GCH ** **+ LBP**	**CDH + ** **CLBP**	**CDH + SHE + ** **CLBP**	**CBRFF [63]**
	0.96	0.90	0.93	0.93	0.94	0.91	0.94	0.95

**Table 6:** Comparison of presented method mAP results versus state of the art methods of 17-Flowers dataset.

17-Flowers Dataset vs. Existing Methods—Mean Average Precision (mAP)									
**Average**	**Proposed Method**	**LCDS [85]**	**SCSPM [86]**	**SCS W/B [86]**	**LCLCIC [87]**	**JLVCD [88]**	**JLVCD W/B [88]**	**FCMOC [89]**	**DLCSPC [90]**
	0.84	0.72	0.52	0.62	0.65	0.69	0.71	0.67	0.59
